# SIRT6 Regulates Protein Synthesis and Folding Through Nucleolar Remodeling

**DOI:** 10.1111/acel.70384

**Published:** 2026-02-17

**Authors:** Daniel Stein, Christian Gallrein, Miguel Portillo, Shai Kaluski‐Kopach, Alfredo Garcia‐Venzor, Yuval Lachberg, Ekaterina Eremenko, Dmitrii Smirnov, Shiran Dror, Monica Einav, Ekaterina Khrameeva, Anat Ben‐Zvi, Björn Schumacher, Debra Toiber

**Affiliations:** ^1^ Department of Life Sciences, Ben‐Gurion University of the Negev Beer‐Sheva Israel; ^2^ The School of Brain Sciences and Cognition Ben‐Gurion University of the Negev Beer‐Sheva Israel; ^3^ Institute for Genome Stability in Aging and Disease, Medical Faculty University and University Hospital of Cologne Cologne Germany; ^4^ Cologne Excellence Cluster for Cellular Stress Responses in Aging‐Associated Diseases (CECAD), Center for Molecular Medicine Cologne (CMMC) University of Cologne Cologne Germany; ^5^ Leibniz Institute on Aging – Fritz‐Lipmann‐Institute (FLI) Jena Germany; ^6^ Center for Molecular and Cellular Biology Skolkovo Institute of Science and Technology Moscow Russia; ^7^ Ilse Katz Institute for Nanoscale Science & Technology Ben‐Gurion University of the Negev Beer‐Sheva Israel

**Keywords:** aging, neurodegeneration, nucleolar expansion, proteostasis, SIRT6

## Abstract

An important hallmark of aging—and particularly of neurodegeneration—is the loss of proteostasis, leading to cellular stress. However, the causal mechanisms driving this loss are unclear. We show that SIRT6 has a critical role in maintaining proteostasis. Mechanistically, SIRT6 negatively regulates global translation by controlling ribosomal genes, nucleolar function and TIP5 chromatin localization. SIRT6 deletion increases nucleolar size, rRNA production and protein translation. However, the expression of chaperones remains unchanged, failing to compensate for the excessive translation, leading to reduced folding capacity and production of aggregates. In vivo, we establish a *C. elegans* model (*sir‐2.4* KO) that shows reduced heat shock resistance and an accelerated age‐dependent reduction in motility. *Sir‐2.4* depletion crossed with a neuron‐specific polyQ strain led to premature motility loss and premature death. These results point to proteostasis‐stress intolerance in the absence of SIRT6, that can be rescued by pharmacologically reducing protein translation rates. Our data suggest that SIRT6 deficiency results in proteostasis loss through nucleolar dysfunction. These results highlight that deficient proteostasis begins with chromatin dysregulation resulting in neurodegeneration.

## Graphical abstract

SIRT6 regulates proteostasis through nucleolar regulation. SIRT6 regulates (A) pre‐rRNA editing through nucleolar factors, as well as (B) rRNA transcription through TIP5 – and thus the NoRC – chromatin recruitment. (C) Once SIRT6 is depleted, the rRNA production and editing are increased, leading to a dramatic elevation in translation rates. However, chaperone levels remain unchanged, leading to the accumulation of misfolded proteins and aggregation upon SIRT6 deletion. (D) In a *C. elegans* ‐based model, deleting the SIRT6 ortholog (*sir‐2.4*) leads to impaired heat shock resistance, as well as reduced motility upon overexpression of a neuronal aggregation‐prone protein (PolyQ40_neuro_ ). (E) However, attenuating translation using 4PBA leads to the rescue of the *sir‐2.* *4*KO;PolyQ40_neuro_ strain.

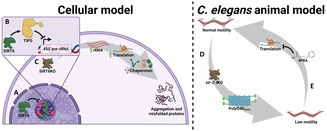

## 1 Introduction

Aging is considered to be a major risk factor for developing a neurodegenerative disease, where the vast majority of the cases are sporadic and age‐related (Prusiner 2012; Bertram and Tanzi 2005). Therefore, understanding the molecular processes that occur during human aging is critical to prevent and treat age‐related neurodegenerative diseases. Neurodegeneration is one of the most predominant age‐related pathologies, with the chances of developing such a condition doubling every 5 years after the age of 65 (Alzheimer's Association 2021).

For more than 30 years the focus in neurodegeneration research has majorly been on the formation of toxic protein aggregates. However, this seems to be a late event in the development of the pathology, and therapies targeting aggregates have failed to improve patients' cognitive capacity and to prevent memory loss (Espay et al. 2019). On the other hand, DNA damage accumulation and epigenetic changes are the key drivers of aging (Soto‐Palma et al. 2022; Schumacher et al. 2021; Toiber and Schumacher 2025), yet their implications on the loss of proteostasis are elusive. In this work, we use SIRT6 as a model of accelerated aging, particularly in the brain, and observe how its loss triggers nucleolar misfunction that results in the loss of proteostasis.

SIRT6 is a nuclear deacetylase and mono‐ADP ribosyltransferase, which plays a critical role in many cellular processes. It represses gene expression through histone deacetylation (Michishita et al. 2008, 2009; Yang et al. 2009) and is involved in the DNA damage response, preventing genomic instability (Mostoslavsky et al. 2006; Toiber et al. 2013; McCord et al. 2009; Mao et al. 2011; Kugel and Mostoslavsky 2014; Onn et al. 2020). SIRT6 is essential especially in mammals: SIRT6 knock‐out (KO) mice show postnatal lethality and genomic instability (Mostoslavsky et al. 2006; Kim et al. 2010; Xiao et al. 2010; Garcia‐Venzor and Toiber 2021). In monkeys, SIRT6 deletion leads to prenatal developmental retardation, delayed neuronal differentiation and to death a few hours postnatally, pointing to a crucial role of SIRT6 in the brain of primates (Zhang et al. 2018). In contrast, SIRT6‐overexpressing mice show increased physical activity and extended lifespan (Kanfi et al. 2012; Roichman et al. 2017). Together, these data suggest that SIRT6 is essential, and its roles are tightly linked to the aging process.

Interestingly, brain‐specific SIRT6‐deficient mice (brSIRT6KO) survive to adulthood but present behavioral defects with major hippocampus‐dependent learning impairments by 4 months of age (Kaluski et al. 2017). Moreover, the brains of these mice show increased signs of DNA damage, cell death, and hyperphosphorylated and acetylated Tau—critical marks in many neurodegenerative diseases (Kaluski et al. 2017; Portillo et al. 2021). Thus, brSIRT6KO phenotypes resemble animal models of Alzheimer's Disease and pathological characteristics of brains from patients suffering from age‐related neurodegeneration (Kaluski et al. 2017). Indeed, SIRT6 levels and activity decrease in aging brains and furthermore in Alzheimer's Disease patients (Kaluski et al. 2017; Portillo et al. 2021; Braidy et al. 2015; Yaku et al. 2018; Fang et al. 2017; Chini et al. 2017). Although neurodegenerative diseases have each a different phenotype, there are many commonalities that we also found when comparing them with SIRT6‐deficient models (Kaluski et al. 2017; Stein et al. 2021). For example, we discovered an additional link between SIRT6 and Alzheimer's, Parkinson's, Huntington diseases and amyotrophic lateral sclerosis (AD, PD, HD and ALS, respectively) through mitochondrial dysfunction (Smirnov et al. 2023). The SIRT6‐neurodegeneration link is especially intriguing because another common trait in many age‐related brain pathologies is the extreme form of proteostasis loss, manifested in aggregation of specific proteins—suggesting a role for SIRT6 in proteostasis. Among these conditions are AD (Ittner and Götz 2011) (with Tau and Amyloid‐beta), PD (Shulman et al. 2011; Dawson and Dawson 2010) (with alpha‐synuclein, parkin and Tau) and HD (Bates 2003; DiFiglia et al. 1997) (with Huntingtin).

While the protein aggregation in AD and PD seems to be a downstream product of a covert age‐dependent malfunction, Huntingtin aggregation in HD is clearly the driver of the disease (Espay et al. 2019). Still, HD develops only as the person gets older and not during development or early adulthood—implying that as the person ages, the capacity to cope with the polyglutamine‐driven aggregation is lost.

Proteostasis includes several layers of regulation ranging from ribosome formation to protein folding and degradation. Translation is done by the ribosomes, which are composed of specialized RNA and protein subunits. While the ribosomal proteins are critical for the regulation of mRNA preference by the ribosome, most of the ribosomal structure—the ribosomal RNA (rRNA)—is expressed and pre‐formed in the nucleolus: a sub‐nuclear membrane‐less compartment, separated by liquid–liquid phase separation (Feric et al. 2016). The nucleolus contains the ribosomal DNA (rDNA) that is transcribed to the 45S pre‐rRNA, which is later processed and assembled—together with ribosomal proteins—into pre‐ribosomal subunits. Importantly, nucleolar physiology is impaired in aging and progerias, with increased nucleolar size and number (Buchwalter and Hetzer 2017; Kriukov et al. 2024). Furthermore, nucleolar size negatively correlates with longevity and its premature enlargement can be seen in progeroid syndromes (Buchwalter and Hetzer 2017; Tiku and Antebi 2018). All these age‐related changes in the nucleolus might lead to altered ribosomal content and translation capacity downstream (Buchwalter and Hetzer 2017). One of the main regulators of the pre‐rRNA transcription is the Nucleolar Remodeling Complex (NoRC), which one of its components is SNF2H—a chromatin remodeler that was previously found to be recruited by SIRT6 to the chromatin at DNA double strand break sites (Toiber et al. 2013).

Here, we reveal the critical role of SIRT6 in maintaining proteostasis from ribosomal production to protein folding. We show that SIRT6 regulates proteostasis through regulation of nuclear and nucleolar genes. SIRT6 negatively regulates translation through the nucleolus: knocking out SIRT6 resulted in enlarged nucleoli; elevated rRNA production; and increased amino acid production and transport to cope with the demand. While this resulted in elevated translation rate, the molecular chaperone family of proteins did not change, leading to reduced protein folding, leaving the cells vulnerable to aggregate formation. Furthermore, we developed a SIRT6‐ortholog KO model in *C. elegans* to show that the role in translation regulation and proteostasis is conserved. We show that the polyQ‐mediated motor neuron dysfunction is exacerbated in *sir‐2.4* KO animals and could be reverted by the FDA‐approved weak translation inhibitor 4‐phenylbutyric acid (4PBA) (Stein et al. 2022). Collectively, these findings establish SIRT6 as a critical regulator of proteostasis through nucleolar activity and emphasize its relevance in aging and neurodegenerative processes.

## 2 Results

### 2.1 SIRT6 Deficiency Affects Pathways of Neurodegeneration, With Common Association to Ribosomal Genes

In order to broaden the insight into the brain function of SIRT6, we inspected our previously published results of brain transcriptomics of brSIRT6KO mouse brains from Smirnov et al. (2023) (Smirnov et al. 2023). These data present 18 significantly downregulated categories in KEGG enrichment analysis, out of which 6 categories belong to neurodegenerative pathologies. Interestingly, however, the most significantly downregulated category was the *Ribosome (ribosomal proteins)* (Figure S1A).

**FIGURE 1:**
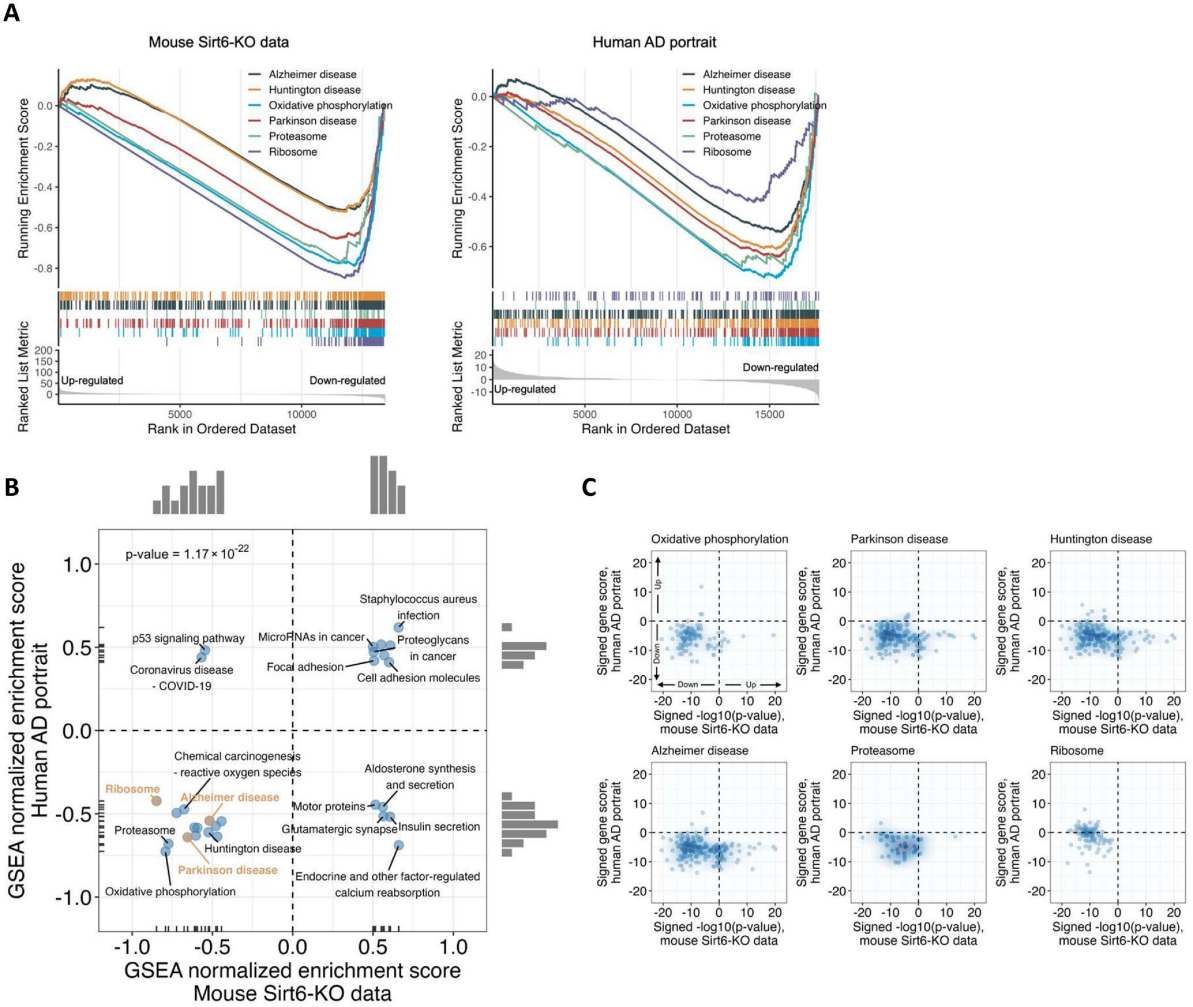
SIRT6 influences aging‐ and proteostasis‐related pathological categories. (A) GSEA plot comparing Alzheimer's disease datasets to brSIRT6KO mouse data, indicating mainly downregulation of these pathways. (B) Correlation plot of genesets enriched in human AD patients and those enriched in brSIRT6KO brains, with colors representing categories as specified in the text. (C) Scatter plot of the signed gene score in AD patients and brSIRT6KO brains, for the selected pathways.

AD is the most common age‐related neurodegenerative disease; since SIRT6 levels decrease in AD (Kaluski et al. 2017), we compared Geneset Enrichment Analysis (GSEA) results of published AD patients' transcriptome portrait (Hill and Gammie 2022) and our brSIRT6KO brains. We found a positive correlation between the two groups of genesets (Figure 1A,B). Again, the *Ribosome (ribosomal proteins)* category as well as *Alzheimer's disease, Huntington's disease*, and *Parkinson's disease* appear to be co‐downregulated in the brains of both AD patients and brSIRT6KO mice, in addition to o*xidative phosphorylation* and *proteasome* categories (Figures 1B and S1B,C). The overlap can be observed in most of the shared sub‐categories (Figure 1C). Interestingly, the protein amount of some significantly downregulated ribosomal proteins revealed that SIRT6 deficiency decouples their mRNA levels from the protein levels (Figure S1D).

Thus, these results emphasize the importance of SIRT6 in both neurodegeneration and ribosomal regulation, as well as the tight correlation between the regulative subunits of the ribosome (namely, the ribosomal proteins) and pathological brain aging.

### 2.2 Lack of SIRT6 Results in Nucleolar Dysregulation and Expansion

**FIGURE 2:**
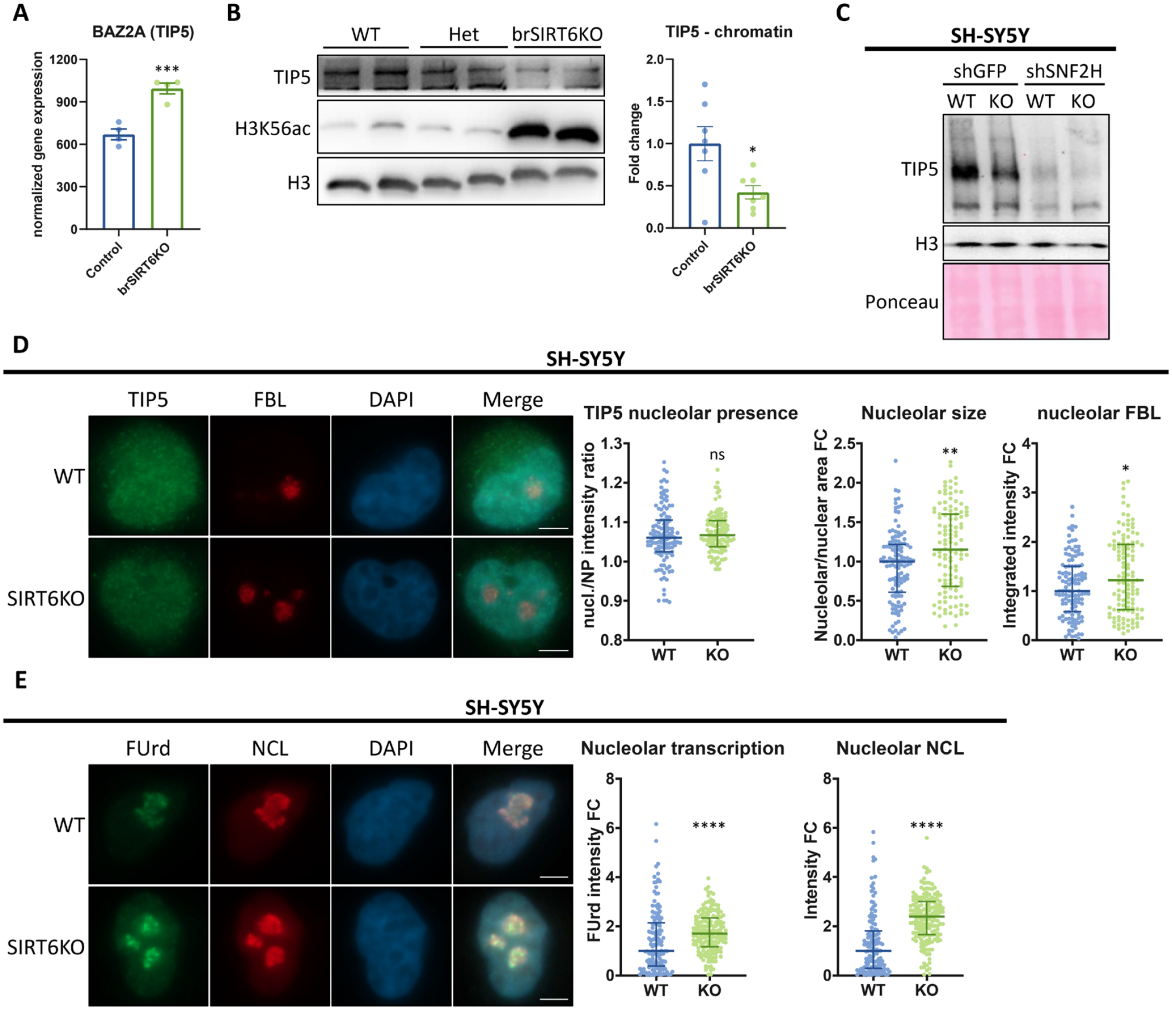
SIRT6 regulates the nucleolar activity. (A) RNA levels of BAZ2A (TIP5 gene) as seen in brSIRT6KO brain RNA‐seq data. (B) A representative western blot of chromatin‐bound proteins from brains of brSIRT6KO, heterozygous (het) and WT mice. H3K56ac – acetylated H3K56 (SIRT6 substrate). Panels (left to right) – representative blots; quantified chromatin‐bound TIP5 intensity, normalized to H3 housekeeping gene. (C) A representative western blot of chromatin‐bound proteins from SH‐SY5Y cells with shSNF2H. WT – control cells; KO – SIRT6KO cells. (D) Immunofluorescence of TIP5 and fibrillarin (FBL) nucleolar marker in control and SIRT6KO SH‐SY5Y cells. Panels (left to right) – representative images; TIP5 nucleolar presence (the ratio between the nucleolar mean intensity and the nucleoplasmic mean intensity); nucleolar fibrillarin (FBL) integrated intensity (sum of all pixel intensities inside the nucleoli); nucleolar size fold change (nucleolar area/nuclear area ratio). *n* = 234. Scale bars = 5 μm. WT – control cells; KO – SIRT6KO cells. Nucl. – nucleolar; NP – nucleoplasm (nucleus excluding the nucleoli); FC – fold change. (E) Immunofluorescence of 5‐fluorouridine (FUrd) and nucleolin (NCL) in SIRT6KO and control SH‐SY5Y cells, that shows the nucleolar transcription. Panels (left to right) – representative photos; quantified FUrd signal inside the nucleolus; nucleolar NCL median intensity. *n* = 296. Scale bars = 5 μm. ns–*p* > 0.05, *p < 0.05, **p < 0.01, ***p < 0.001, ****p < 0.0001.

SIRT6 could be involved in ribosomal changes also through its known interaction with SNF2H (Toiber et al. 2013) and the role of the latter in the Nucleolar Repressive Complex (NoRC). This complex is directed at the rDNA by its largest component: TIP5 (also known as BAZ2A). While TIP5 RNA levels are increased in brSIRT6KO mice brains (Figure 2A), its total protein levels, as well as chromatin recruitment, are both reduced (Figures 2B and S2A,B). Consistently, TIP5/BAZ2A was previously found to be induced in human AD patients' brains (Gallrein et al. 2023). When observing SNF2H—a chromatin remodeling subunit of NoRC—the brSIRT6KO brains show no significant difference in total protein levels, while its chromatin recruitment has a clear reduction, as expected by previous reports (Toiber et al. 2013) (Figure S2A,B). Moreover, SNF2H knockdown dramatically reduces TIP5 chromatin binding, and the combination with SIRT6KO reduced it slightly more (Figure 2C). These results reveal that SNF2H is cardinal to NoRC rDNA binding, and that while both SIRT6 and SNF2H are important to TIP5 recruitment, they might have additional distinct pathways to do so. Interestingly, when measuring TIP5 nucleolar presence—i.e., the ratio between TIP5 in the nucleoli and TIP5 in the nucleoplasm (nucleus without nucleoli)—it does not change in SIRT6KO cells (Figure 2D). Thus, TIP5 is not recruited to chromatin due to NoRC dysregulation, and not due to the absence of TIP5 from the nucleolar region.

A reduced NoRC recruitment to chromatin could lead to derepression at rDNA loci and possibly increase pre‐rRNA production. Therefore, we tested nucleolar transcription with 5‐fluorouridine (FUrd) labeling and found that SIRT6‐deficient cells have elevated rRNA production in the nucleolus (Figure 2E). These results suggested that SIRT6 regulates ribosomal RNA transcription.

**FIGURE 3:**
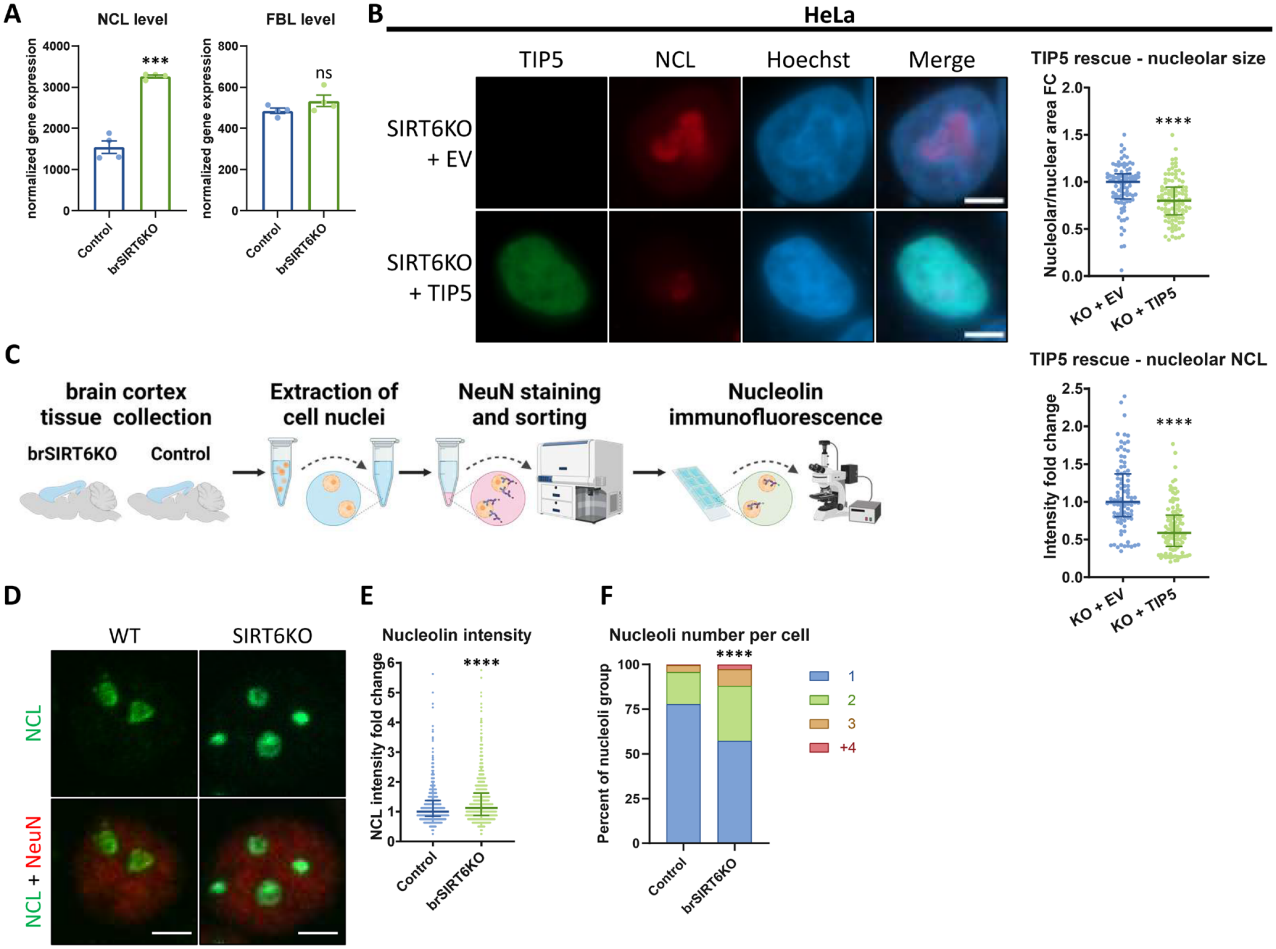
Nuclear expansion in SIRT6 deficiency. (A) RNA levels of Nucleolin (NCL) and Fibrillarin (FBL), as seen in brSIRT6KO brains RNA‐seq data. (B) Immunofluorescence of the nucleolar marker nucleolin (NCL) in TIP5‐overxpressing SIRT6KO and control HeLa cells. Panels (upper left to lower right) – representative photos; nucleolar size fold change (nucleolar area/nuclear area ratio); quantified nucleolar nucleolin (NCL) median intensity. *n* = 193. Scale bars = 5 μm. WT – control cells; KO – SIRT6KO cells; EV – empty vector. (C) A schematic representation of NCL staining in NeuN‐positive nuclei isolated from mouse cortices. (D) Immunofluorescence of NCL (green) and NeuN (red) in isolated nuclei of brSIRT6KO and control mouse brain neurons. *n* = 9 mice. Scale bars = 5 μm. (E) Quantification of NCL intensity in isolated NeuN‐positive nuclei of brSIRT6KO mice (Figure 3C,D). (F) Percentage of nucleoli number in each NeuN‐positive nucleus, in brSIRT6KO mouse brain cortices (Figure 3C,D). ns–*p* > 0.05, ***p < 0.001, ****p < 0.0001.

Since nucleolar size and function are established biomarkers of aging, we hypothesized that reduced NoRC regulation and increased nucleolar transcription upon SIRT6 deficiency could affect nucleolar size and function. We assessed the key nucleolar markers Nucleolin and Fibrillarin (NCL and FBL, respectively), which are critical for nucleolar rRNA post‐transcriptional processing. In brSIRT6KO mouse brains, we saw a significant increase of the mRNA levels of NCL but not of FBL (Figure 3A). Next, we tested our SIRT6‐deficient cellular models. In SIRT6KO SH‐SY5Y, we find increased total nucleolar area per nucleus, as well as elevated median pixel intensity of NCL—meaning overall brighter NCL signal per area unit (Figure 2D,E). In FBL, on the other hand, the integrated intensity (the total intensity sum) is significantly increased, but not the median pixel intensity (Figures 2D and S2C). The elevation in NCL median intensity is notable, as it suggests that the abnormal nucleolar activity stems from altered nucleolar homeostasis and higher NCL density, and not merely due to nucleolar expansion. Moreover, these results were largely recapitulated in HeLa cells (Figure S3A), but with elevated FBL median intensity too—suggestive of a cell line‐specific effect on FBL. Importantly, we overexpressed TIP5 in SIRT6KO HeLa cells, and this significantly reduced the nucleolar expansion and elevated NCL activity—validating our model: the observed nucleolar abnormalities in SIRT6 deficiency are indeed TIP5/NoRC dependent (Figure 3B). Collectively, these data indicate that SIRT6 deficiency drives not only rRNA overproduction, but also its processing in the nucleolus.

To confirm that the nucleolar abnormalities take place in post‐mitotic neurons, we isolated nuclei from NeuN‐positive cortical cells of adult WT and brSIRT6KO mice (9.5–13 months old; see scheme on Figure 3C). Neurons from brSIRT6KO mice presented NCL overexpression (Figure 3D,E). Importantly, the number of nucleoli per nucleus followed a similar trend: brSIRT6KO brains present significantly more nucleoli than their WT counterparts (Figure 3D,F). Thus, our results in cell lines are also recapitulated in cortical neurons of adult mice.

Our combined results from mouse brains and different cell lines support the notion that lack of SIRT6 and aging compromise the nucleolus by affecting pre‐rRNA synthesis and processing, together with nucleolar function and physiology. This, in turn, plays an important role in the nucleolar expansion and overproduction of rRNA. Importantly, combining the dramatically elevated rRNA production with no corresponding elevation in ribosomal protein quantities of similar magnitudes potentially points to abnormal regulation of translation at the ribosome level.

### 2.3 SIRT6‐Deficient Cells Have Increased Protein Synthesis

**FIGURE 4:**
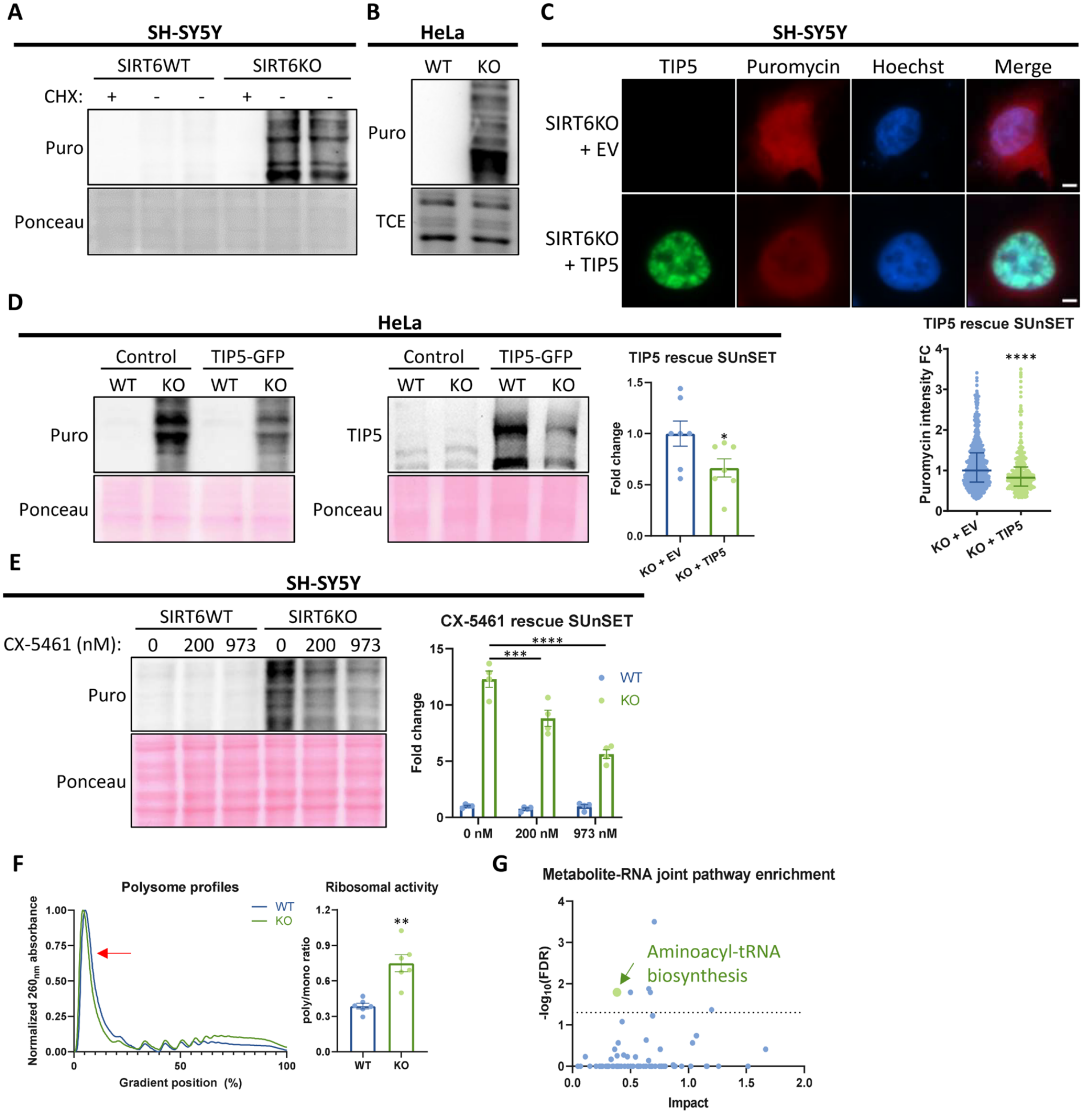
SIRT6KO results in hyper‐translation. (A, B) Representative western blots of puromycin‐based nascent protein labelling (SUnSET), in (A) SH‐SY5Y and (B) HeLa. WT – control cells; KO – SIRT6KO cells; Puro – puromycin; CHX – cycloheximide (negative control). (C) Immunofluorescence of puromycin‐labelled nascent proteins in TIP5‐overexpressing SIRT6KO SH‐SY5Y cells. Panels (top to bottom) – representative photos; quantified puromycin intensity. *n* = 1028. Scale bars = 5 μm. WT – control cells; KO – SIRT6KO cells; EV – empty vector. (D) A representative western blot of nascent protein labelling (SUnSET) in HeLa cells overexpressing TIP5‐GFP. Panels (left to right) – representative blots; quantified puromycin intensity, normalized to Ponceau staining. (E) A representative western blot of nascent protein labelling (SUnSET) in SH‐SY5Y cells treated with CX‐5461 (RNA Polymerase I inhibitor) for 24 h. Panels (left to right) – a representative blot; quantified puromycin intensity, normalized to Ponceau. (F) Ribosome profile analyses of SIRT6KO and WT HeLa cells. Red arrow indicated the monosome peak. Left panel – representative profiles; right panel – polysome/monosome ratio analysis. (G) A pathway enrichment analysis for combined metabolome and differentially expressed genes of brSIRT6KO brains. Dotted line – FDR = 0.05. *p < 0.05, **p < 0.01, ***p < 0.001, ****p < 0.0001.

We suspected that the enhanced production and processing of rRNA could lead to elevated translation. To test this in our models, we directly measured translation using the SUnSET technique (Schmidt et al. 2009). We found an increased protein synthesis rate in SIRT6KO SH‐SY5Y, HeLa and HEK293T cells (Figures 4A,B and S4A,B). To validate that the SIRT6KO hyper‐translation is mediated by nucleolar dysregulation, we repeated the SUnSET assays with TIP5 overexpression and found that indeed, it partially rescues the elevated translation rates in SIRT6KO SH‐SY5Y and HeLa cells (Figure 4C,D). However, the rescue effect is relatively mild compared to the basal translation rates, probably due to low transfection efficiency, especially in SIRT6KO cells, and probably since the overexpression only partially compensates for the reduced capacity to recruit TIP5 protein (and hence NoRC) to the chromatin. The enzyme that transcribes the rRNA in the nucleolus is RNA polymerase I (Pol I), so we measured protein synthesis rates in CX‐5461 treatments (Pol I‐specific inhibitor) and found again that it rescues translation in SIRT6KO SH‐SY5Y cells (Figure 4E). We could not detect any significant effect in WT cells, supporting the importance of pre‐rRNA synthesis in SIRT6KO‐dependent hyper‐translation. Thus, our data suggest that the nucleolar dysregulation resulting from SIRT6 deficiency leads downstream to elevated rRNA and protein biosynthesis, and either overexpressing TIP5 or directly reducing rRNA synthesis rescues the hyper‐translation.

We then performed polysome profiling for SIRT6‐deficient HeLa cells and found there is a clear increase in polysome/monosome ratio upon SIRT6KO, pointing to elevated ribosomal activity (Figure 4F). Previously, Ravi et al. (2019) (Ravi et al. 2019) found that SIRT6‐deficient cardiac tissue had increased translation due to misregulation of the translation initiation regulator 4EBP1. Intriguingly, the elevated translation we observed was independent of translation initiation regulators: we measured 4EBP1 phosphorylation and found no difference in SIRT6KO cells (Figure S4C), in contrast to Ravi et al. (2019) (Ravi et al. 2019). Furthermore, we measured eIF2α activity in brains deficient of SIRT6, but there was no significant effect as well (Figure S4D). This suggests that mechanistically, the translation initiation factors are not responsible for the changes observed in translation rates in the brain, but rather the elevated ribosomal content.

To support the increased protein synthesis upon SIRT6 deficiency, the intracellular amino acid content should be elevated. The brSIRT6KO brain RNA‐seq data reveals that indeed, there is a significant increase in many amino acid transporters that together cover all the amino acids (Figure S4E). Analyzing brSIRT6KO brain metabolomics revealed that out of the top 25 enriched categories, 10 were related to amino acid and protein metabolism (Figure S4F, marked in red arrows)—pointing to the dramatic metabolic changes that occur in SIRT6‐deficient brains. We then performed a joint pathway enrichment analysis by integrating the RNA‐seq data with metabolomics data, using the Pathway Analysis tool on MetaboAnalyst. This analysis revealed six significantly altered pathways (FDR < 0.05). One of them is ‘Aminoacyl‐tRNA biosynthesis’ (Figures 4G and S4G). Analyzing SIRT6KO SH‐SY5Y cells metabolomics supported the mouse data, as intracellular content was significantly elevated for most detected amino acids (Figure S4H). We conclude that the hyper‐translation in SIRT6 deficiency is supported by a metabolic shift in amino acid content and is driven by elevation in polysomes.

### 2.4 SIRT6 Deficiency Impairs Protein Refolding

**FIGURE 5:**
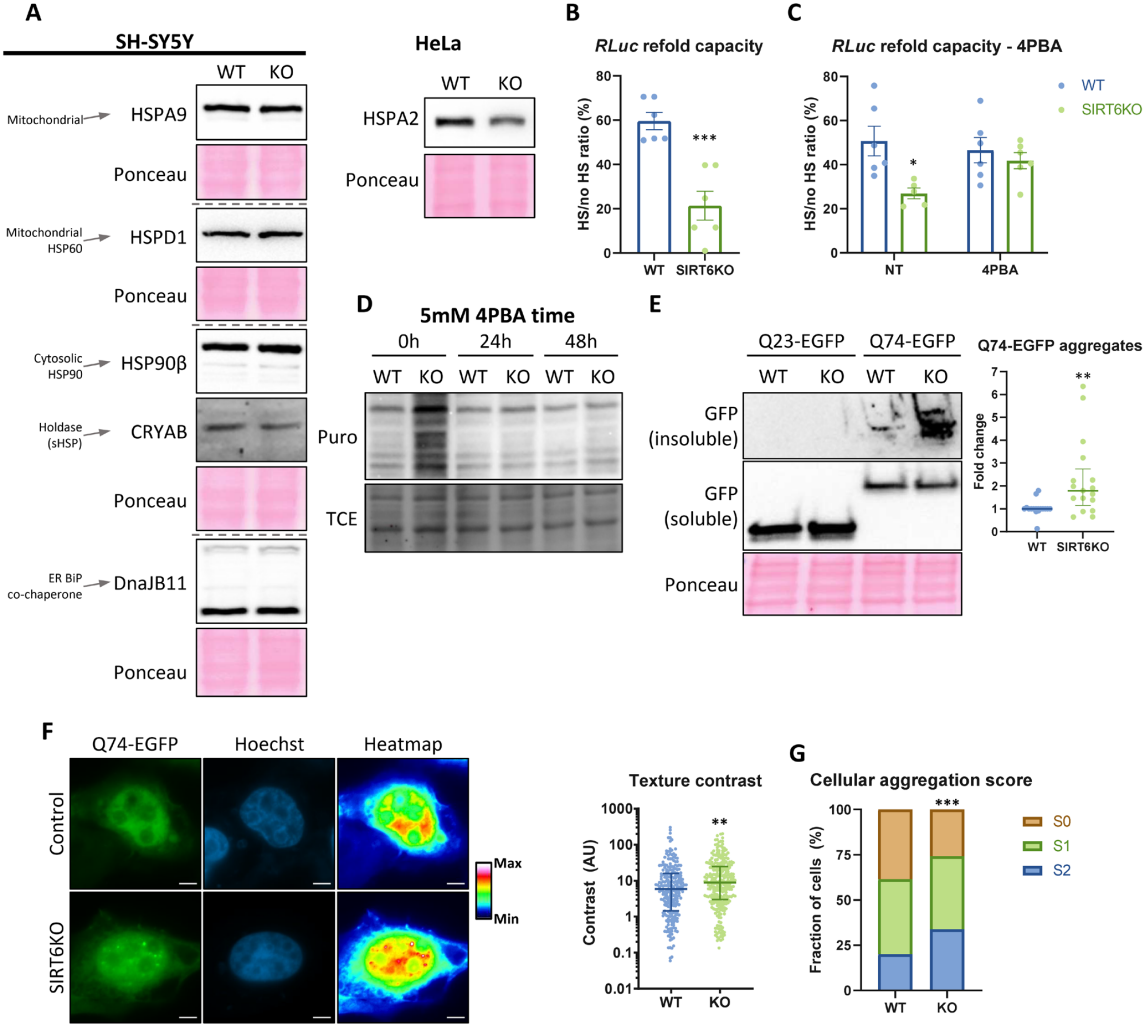
SIRT6KO impairs protein folding. (A) representative western blot of chaperones in SIRT6KO cells. WT – control cells; KO – SIRT6KO cells. (B, C) *Renilla* Luciferase protein refold capacity after heat shock, in HEK293T cells with either (B) no treatment or (C) 4PBA‐based translation attenuation. Y axes – the fraction of active protein, compared to the normal level in non‐heat‐shocked cells. NT – no treatment. 4PBA – 48 h 5mM 4PBA. (D) A representative western blot of SUnSET nascent protein labelling in HEK293T cells, treated with 4‐phenylbutyric acid (4PBA). (E) A representative western blot of Q23/Q74‐EGFP expression and aggregation in SH‐SY5Y cells, with normalized quantifications. (F) Representative photos of Q74‐EGFP expression pattern in SH‐SY5Y cells. Heatmap – Q74‐EGFP intensity was altered to heatmap LUT to emphasize differences in aggregation and texture among cells. Right panel – distribution of cells based on their texture (smoothness/roughness), representing aggregation. Scale bars = 5 μm. (G) Manual scoring of cells based on the Q74‐EGFP aggregation. S0 – no aggregates/smooth cell texture; S1 – 1–5 aggregates/medium cell texture; S2 – more than 5 aggregates/rough cell texture. *p < 0.05, **p < 0.01, ***p < 0.001.

In order to be functional, proteins must be properly folded. As folding is very delicate, it often requires the aid of a specialized group of proteins—molecular chaperones (Saibil 2013). While our results show elevated protein synthesis rates, chaperones and folding machinery were lacking in the significantly changing gene categories (Figure S1A). Therefore, we manually inspected the brSIRT6KO RNA‐seq for proteostasis categories and found that the 33 proteostasis‐ and protein folding‐related categories in the analysis were not changed significantly (Figure S5A). Indeed, when verifying the protein levels of several chaperones, no considerable increase was found, even though various translation‐ and protein folding‐related stresses are usually accompanied by a considerably increased chaperone machinery (Figure 5A). Thus, we could not detect any systemic change in chaperone network.

We speculated that while there is a large mass of newly translated proteins, the support of the folding machinery is insufficient, rendering the accumulation of misfolded proteins. To test that, we measured the cellular capacity of refolding misfolded proteins, using a luciferase‐based heat shock‐recovery assay: Luciferase is very sensitive to heat, and inducing a heat shock reduces its activity. Cells with competent proteostasis machinery will manage to refold it over time—a property measured by its activity (see Figure S5B for a scheme). Our results indicate that while HEK293T WT cells managed to recover ~60% of *Renilla* Luciferase activity, SIRT6KO cells could recover only ~20% (Figure 5B). To validate that the reduced recovery is driven by the hyper‐translation, we treated the cells with a translation attenuator. We previously demonstrated that 4‐phenylbutyric acid (4PBA) is a conserved protein synthesis inhibitor, and contrary to the literature—has little or no effect as a chemical chaperone (Stein et al. 2022). In concert with our hypothesis, translation attenuation helped to fully recover the folding capacity of the SIRT6KO cells (Figure 5C). As expected, this treatment reduced the translation rate in SIRT6KO cells to similar levels as WT in all tested cell lines (Figures 5D and S5C–E), further supporting that the 4PBA rescue is through translation attenuation (Stein et al. 2022).

To conclude, the hyper‐translation upon SIRT6KO is not accompanied by a proper folding‐promoting environment in the cell, which results in a low capability to properly fold newly translated proteins or to refold misfolded proteins—potentially leading to deleterious effects on the cell.

### 2.5 Lack of SIRT6 Results in Increased Protein Aggregates

In normal conditions, persistently un‐ or misfolded proteins go to degradation; if not, they might accumulate into toxic insoluble aggregates, as often seen in neurodegeneration (Sweeney et al. 2017). Normally, due to their size, protein aggregates are degraded through autophagy—a potential fate for the massively accumulating misfolded proteins in SIRT6KO. Therefore, we measured the autophagy marker LC3 in brains and cell lines under normal conditions or stress, but no differences were found (Figure S5F,G).

To further understand the role of SIRT6 in aggregation, we used an aggregation‐prone polyglutamine (polyQ) vector fused to EGFP (Q74‐EGFP), which is based on the structural motif in the aggregation mechanism in HD. Briefly, HD patients have CAG trinucleotide repetitions in the HTT gene, which give rise to a polyQ chain in the translated protein N‐terminus. Unlike healthy individuals, HD patients have more than 35 consecutive repeats, which form β‐sheet structures that tend to aggregate in cells and become toxic; the longer the polyQ chain—the harsher and earlier the pathology appears (Jiang et al. 2023).

First, we measured aggregation in SIRT6KO SH‐SY5Y cells. SIRT6‐deficient cells have increased levels of Q74‐EGFP aggregates that cannot migrate through the SDS gel and hence are stuck in the wells, compared to WT controls (Figure 5E). Thus, the hyper‐translation upon SIRT6KO is also accompanied by increased aggregate formation.

Next, we observed the EGFP‐positive cells in a microscope and measured the Q74 aggregation through the texture of the EGFP signal (how smooth/rough the texture is), by calculating the contrast between neighboring pixels in an unbiased manner (see the Section 4.15 in *Methods* for more details). We found a significant increase in texture contrast in SIRT6KO (Figure 5F), indicative of more aggregation upon SIRT6 deficiency.

We also classified manually the EGFP‐positive cells into 3 score groups: S0—no foci nor granulation; S1—up to 5 foci or medium granulation; S2—more than 5 foci or heavily granulated cells. Our data show that SIRT6KO leads to a larger portion of S2 on the expanse of the S0 population (Figure 5G).

### 2.6 *C. elegans Sir‐2.* *4*KO Model Recapitulates the Role of SIRT6 in Proteostasis

**FIGURE 6:**
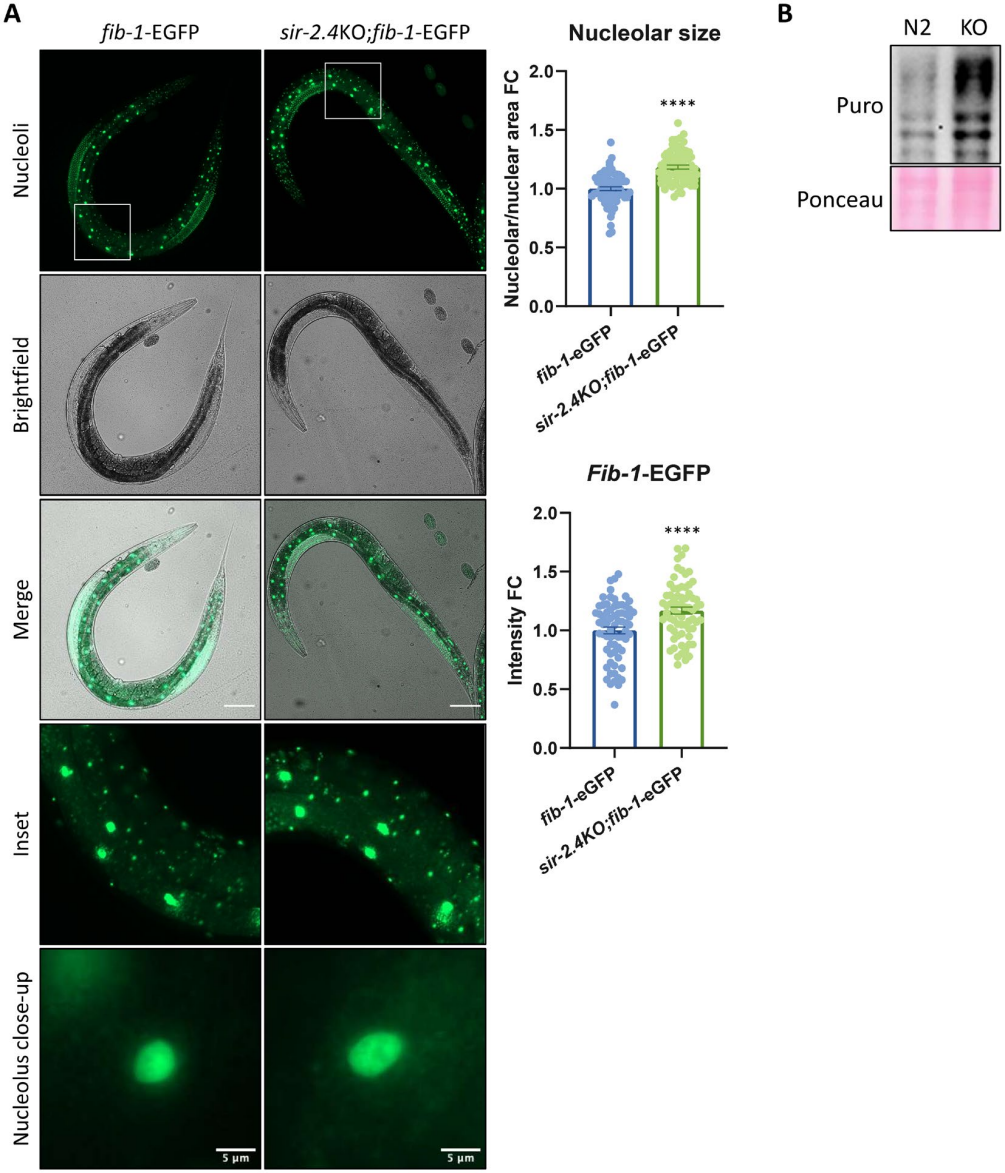
A SIRT6 ortholog KO model in a whole organism recapitulates the nucleolar abnormalities. (A) *Fib‐1*‐EGFP‐based visualization of the nucleoli in *C. elegans*, including quantifications of the nucleolar area and *fib‐1*‐EGFP intensity. *Fib‐1* – fibrillarin. *n* = 137 nuclei, 69 animals. Scale bars of the full worm = 100 μm. (B) A representative western blot of nascent protein labelling (SUnSET) in *C. elegans*. N2 – control strain; KO – *sir‐2.4*KO strain; Puro – puromycin. ****p < 0.0001.

To test the effects of SIRT6 on proteostasis in a full physiological context, we developed a new SIRT6‐deficient model in *C. elegans* by knocking out *sir‐2.4*—SIRT6 ortholog in nematodes (*sir‐2.4*KO; Figure S6A,B). To measure their nucleoli, these worms were crossed with the *fib‐1‐*EGFP strain (fibrillarin nucleolar reporter strain). In concert with our previous results, these worms present nucleolar expansion and elevated nucleolar activity, and downstream increased translation rates compared to the corresponding genetic background strains (Figure 6)—recapitulating our results in cell lines.

**FIGURE 7:**
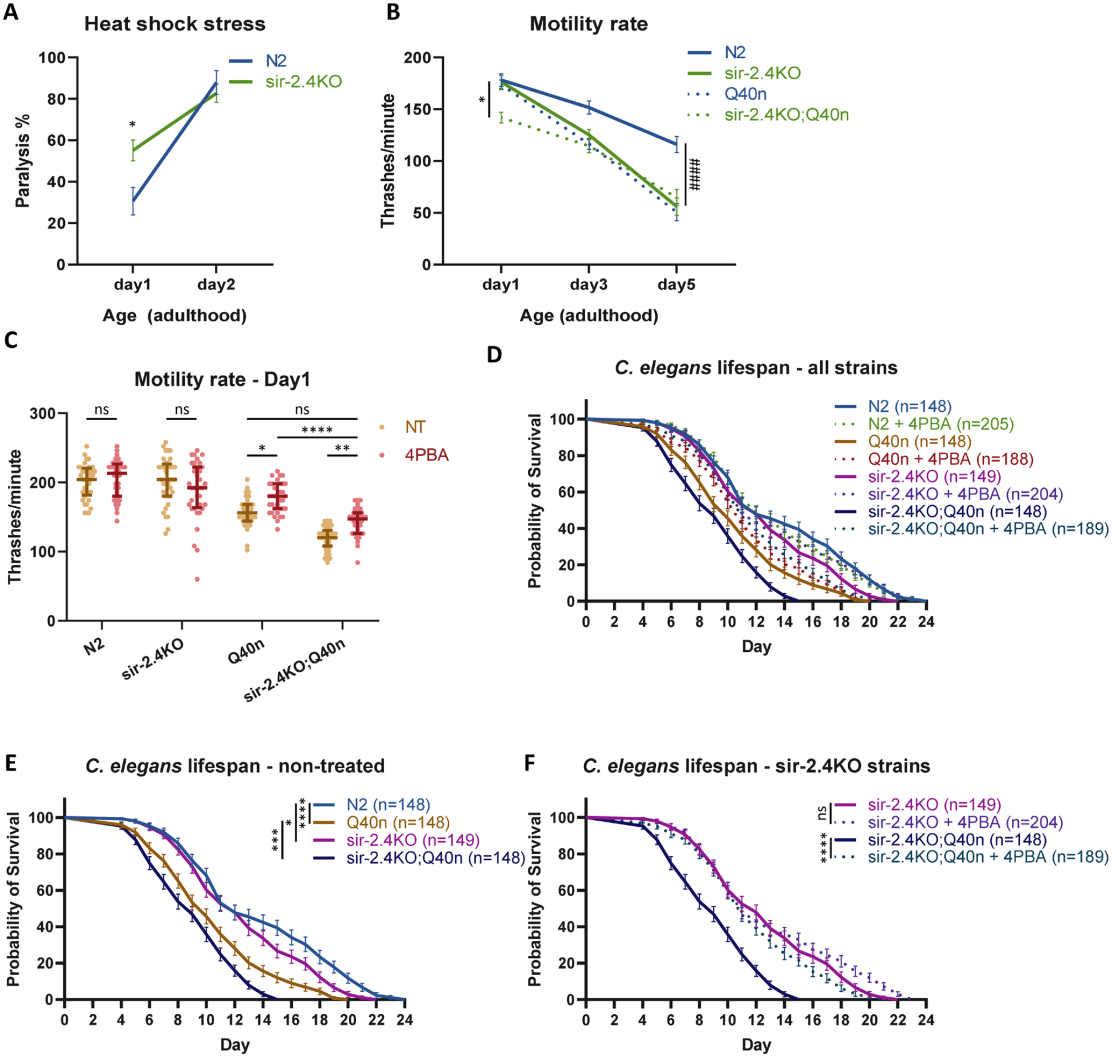
*Sir‐2.4* KO deteriorates proteostasis in *C. elegans*. (A) Heat shock resistance assay, measuring the paralysis rate after a heat shock in days 1 and 2 of adulthood, followed by an overnight recovery period. (B) Motility rate assay, measuring the aging progression in days 1, 3, 5 of adulthood, both in the basal strains (N2/*sir‐2.4*KO) and the Q40n‐crossed strains. ####*p* < 0.0001 between N2/*sir‐2.4*KO strains; *p < 0.05 between the Q40n‐crossed strains. (C) Motility assay with 1.65mM 4‐PBA from L4 stage to day 1 of adulthood. (D) Survival assay data for all *C. elegans* strains. (E, F) selected individual strains and treatments, separated for clarity. ns–*p* > 0.05, *p < 0.05, **p < 0.005, ****p < 0.0001.

To test proteostasis capacity in vivo, we stressed the nematodes with a heat shock and measured paralysis after 24 h of recovery. We found that the N2 (genetic background WT) worms presented, as expected, a heat shock‐driven increase in paralysis only after the reported collapse of proteostasis at day 2 of adulthood, from ~30% to ~88% paralyzed worms (Figure 7A) (Shemesh et al. 2013). However, the *sir‐2.4*KO worms showed a poor resistance to heat shock even at day 1 of adulthood—with ~55% paralysis before the expected collapse of proteostasis (Figure 7A). These data suggest that the roles of SIRT6/*sir‐2.4* in translation regulation and proteostasis maintenance are highly conserved, and loss of proteostasis occurs prematurely in the *sir‐2.4*‐deficient animals.

Moreover, we conducted a thrashing‐based motility assay and found that the KO strain presented a more rapid age‐dependent reduction in motility rate compared to WT (Figure 7B, full lines), suggestive of a faster decline of motor neuron function in *sir‐2.4* deletion. To further elucidate *sir‐2.4*'s role in neuronal proteostasis specifically, we adopted a polyQ‐based proteostatic stress model in nematodes: we crossed the *sir‐2.4*KO worms with a neuron‐specific polyQ‐expressing strain with 40 glutamine repeats (Q40n). We found an earlier collapse of motility compared to the genetic background‐matching strain (Figure 7B, day 1, dotted lines). Interestingly, both strains reached similar motility rates on Day 3 of adulthood. These data suggest that in *sir‐2.4* deficiency, loss of proteostasis becomes harsher and manifests a deteriorated neurodegenerative phenotype and premature aging.

Next, we tested the ability of translation attenuation to rescue the Q40n motility impairment using 4PBA treatment. Since *sir‐2.4*KO affects proteostasis‐stressed worms already at day 1 of adulthood, we started the 4PBA treatment at the last larval stage 4 (L4) preceding adulthood (Figure 7A,B). Importantly, we first verified that 4PBA has the same translation attenuation effect in *C. elegans* as we previously showed in other models (Stein et al. 2022) (Figure S7A,B). WT and *sir‐2.4*KO strains presented no effect in 4PBA treatment, as both these strains are before the expected proteostasis collapse (Figure 7C). On the other hand, 4PBA translation attenuation significantly reduced the Q40n‐YFP protein levels in both Q40n and *sir‐2.4*KO;Q40n strains (Figure S7C), and as a result alleviated the motility decline in these strains (Figure 7C). This pinpoints the critical role of translation rates in polyQ aggregation. Moreover, 4PBA rescued the *sir‐2.4*KO;Q40n worms nearly to the level of the non‐treated Q40n strain, albeit not to the degree of the 4PBA‐treated Q40n worms (Figure 7C). Thus, these results support our hypothesis that the proteostatic collapse upon *sir‐2.4* deficiency is driven by the hyper‐translation. These results indicate that pharmacological reduction of protein synthesis could alleviate polyQ‐related decline of neuronal function.

Finally, we conducted a lifespan assay and observed that the deletion of *sir‐2.4* has a mild negative impact on the lifespan of N2 (WT) worms (Figure 7D,E, S7G). In polyQ worm strains, the Q40n expression reduces lifespan as expected, and adding the deletion of *sir‐2.4* to the Q40n reduces lifespan even further—indicating the increased sensitivity to Q40n accumulation in *sir‐2.4*‐deficient neurons (Figure 7D,E, S7E,G).

Notably, 4PBA treatment had no significant effect on the lifespan of the N2 and *sir‐2.4*KO worms, but on the other hand it had a mild improvement in Q40n and a very dramatic effect in *sir‐2.4*KO;Q40n mutants (Figure 7D,F, S7D–G). These results suggest that the translation attenuation has a limited rescue effect on the long‐term proteostatic impairment exerted by aggregated proteins alone. However, when the *sir‐2.4*KO nucleolus‐driven hyper‐translation is modulated with 4PBA, the aggregated Q40n effect is dramatically restrained—again, supporting our SIRT6‐nucleolus‐translation model for proteostasis loss.

Taken together, our results establish that SIRT6 has a highly conserved function in regulating proteostasis by controlling nucleolar gene expression. Once chromatin is more open and allows the expression of ribosomal genes and translation‐required machinery, the cells enter a pathological mode of hyper‐translation with no enhancement in protein folding capacity. The ensuing accumulation of misfolded proteins and aggregates becomes toxic to the neurons, impairing movement of the organism (Van Pelt and Truttmann 2020). Thus, our data suggest that the loss of SIRT6, that occurs naturally upon aging, provokes increased protein synthesis but reduces protein folding capacity—thus leading to pathological aging and neurodegeneration.

## 3 Discussion

Loss of proteostasis is a hallmark of aging and neurodegenerative diseases. Still, in the absence of mutations that result in protein aggregation, it is poorly understood why proteostasis capacity is diminished in aging. To the best of our knowledge, we are the first to show that loss of SIRT6, as it occurs in aging and neurodegeneration, is causal of the proteostasis loss by altering the balance between translation and folding capacity of the cells. Moreover, the SIRT6 reduction can also explain the appearance of nucleolar expansion and aggregates in aging and neurodegeneration.

Decreased levels of SIRT6 disrupt the recruitment of chromatin remodelers and regulators, resulting in elevated transcription and imbalanced gene expression, particularly within the nucleolus. In addition to the elevated nucleolar transcription, the nucleolar rDNA repeats are also especially prone to DNA damage. Consequently, upon SIRT6 deficiency, the elevated transcription rates—coupled with nucleolar expansion—might enhance the vulnerability to DNA breaks. Furthermore, compromised DNA repair mechanisms in the absence of the sensor SIRT6 (Onn et al. 2020) perpetuate a harmful cycle of DNA damage, nucleolar expansion, and increased nucleolar transcription. This cycle significantly impacts protein synthesis and homeostasis both in quantity, by affecting ribosome production, and in quality, by the introduction of mutations.

We observed that SIRT6 regulates not only cap‐dependent translation as previously reported (Ravi et al. 2019; Ben Lulu et al. 2025), but the entire regulation of ribosomes. Upon SIRT6 deletion, the rRNA synthesis and processing are elevated, but with no concomitant elevation in ribosomal protein genes, interfering with the stoichiometric balance between these ribosomal components. We speculate that this leads not only to the dramatic elevation in total translation rates as we observe here (as there are, in total, more ribosomes) but also to dysregulation in which transcripts to translate by the ribosomes, hence affecting the efficiency, regulation, and mRNA specificity of translation. The exact effect on transcript choice in SIRT6 deficiency, however, remains to be determined.

Although translation is increased in SIRT6 absence, the chaperone‐ and protein folding‐related genes nearly do not change, leading to a deteriorated ability to properly fold proteins—a critical machinery in every cell. Importantly, the polyglutamine models reveal that upon SIRT6 deficiency, aggregation‐prone stress results in higher aggregation. These roles of SIRT6 in proteostasis are well conserved from nematodes to humans.

As we demonstrated, the observed reduced proteostasis is rescuable using translation attenuation, by allowing the system to regain the balance between protein synthesis and folding machinery. Importantly, the efficacy of the FDA‐approved drug 4PBA suggests a clinically implementable pharmacological strategy to alleviate SIRT6 deficiency‐driven protein misfolding conditions, whether in SIRT6‐deficient patients or during the normal decline of SIRT6 during aging.

The role of SIRT6 in regulating proteostasis is evolutionarily conserved—its *C. elegans* ortholog, *sir‐2.4*, also has a critical role in translation and maintaining thermal resistance and motility in worms as they age. In reproducing nematodes, proteostasis is known to collapse between day 1 and day 2 of adulthood (Shemesh et al. 2013). Here, we show that combining *sir‐2.4* deletion and proteostatic stress precedes the collapse to day 1, pinpointing its critical role in proteostasis and health. Moreover, Taylor et al. (2022) showed in *D. melanogaster* that overexpressing dSIRT6 reduces translation rates, and Kaluski‐Kopatch et al. (2025) showed a change in gene categories related to ribosome biogenesis, PD and ALS in dSIRT6‐deficient flies—further strengthening our results. From another evolutionary perspective, the different aspects of ribosome biogenesis and function can be coregulated by several master regulators—one of them is SIRT6.

While DAF‐16/FoxO has been linked to *sir‐2.4*‐mediated stress response in *C. elegans* (Chiang et al. 2012), our findings suggest a distinctive mechanism in the context of protein folding stress. FoxO typically regulates protein synthesis through the mTOR pathway and activates 4EBP1, yet we observed no 4EBP1 changes in our cells. Additionally, we detected no significant phosphorylation changes in eIF2α in SIRT6KO cells or brains, nor any alterations in cleaved or total LC3 levels associated with autophagy. Instead, increased translation—reduced by 4PBA—appears to drive the observed sensitivity in *sir‐2.4* KO worms, impacting longevity and pointing to a distinct regulatory pathway. Moreover, Jedrusik‐Bode et al. (2013) have shown SIRT6 to appear in stress granules after stress, adding to the regulation of proteostasis another layer.

Previous work found that DNA damage leads to a temporary transcription‐translation stalling, followed by an increased protein synthesis after DNA damage was repaired—lowering the chance of accumulating mutated RNA and proteins (Wang et al. 2020). Here, we see that SIRT6KO leads to accelerated translation, bypassing this important regulatory mechanism of translation attenuation, even though DNA damage is known to be elevated in SIRT6 deficiency (Mostoslavsky et al. 2006; Kaluski et al. 2017). We speculate that SIRT6 deletion is so deleterious to the organism, precisely because of the harsh imbalance it causes: SIRT6‐deficient cells are in a state of chronic DNA damage, but as SIRT6 is a critical sensor of DNA double strand breaks, its absence impairs the repair (Onn et al. 2020). Moreover, the absence of SIRT6 compromises also its role as a gene regulator, leading to epigenetic dysregulation and to nucleolar hyperactivity. Thus, the combination of DNA damage and hyper‐translation could also result in the synthesis of mutated RNA molecules (from damaged DNA molecules), which further pushes the loss of proteostasis apparent in SIRT6KO. As SIRT6 activity declines with normal aging, and even further in AD patients, this implies a mechanism which feeds the loss of proteostasis—in several ways—in aging and age‐related brain pathologies.

We show elevation in protein synthesis in several models and techniques (Figures 4A–F, 5D, 6B, S4A,B, and S5C–E, S7A,B). However, viewing the currently translated proteins using microscopy revealed considerable nuclear signal, suggestive of diffusion and trapping of small puromycylated nascent peptides through the nuclear envelope. While this may be a limitation of the method, as suggested by previous reports (Enam et al. 2020), it may also indicate the potential labeling of nuclear‐diffusing defective ribosomal products (DRiPs) (Mediani et al. 2019)—which are related to ribosomal errors or defects in the translation process. This can be of particular interest in the context of ubiquitin‐proteasome system‐related degradation; however, it is outside the scope of this work.

Another limitation in our data is the measurement of the endogenous protein aggregation rate in the absence of SIRT6. Both the polyQ‐ and Luciferase‐based methods use exogenous proteostasis stressors and do not address the basal level of aggregation/protein misfolding in SIRT6KO. Regrettably, technical limitations in quantifying these native aggregates prevent us from drawing definitive conclusions regarding basal proteostatic stress in SIRT6 deficiency.

To conclude, we show a novel role of SIRT6 in nucleolar regulation and proteostasis. This role explains how loss of proteostasis begins with nucleolar dysregulation, and how this regulation widely affects translation and proteostasis downstream, leading to the aggregates seen in age‐related neurodegenerative diseases. We suggest that the FDA‐approved 4PBA could provide a clinically accessible route for reversing the protein folding stress by mildly reducing protein translation and subsequently alleviating the neurodegenerative consequence of protein aggregates.

## 4 Methods

### 4.1 Generation of Brain‐Specific SIRT6KO Mice and SIRT6KO Cells

Brain‐specific SIRT6KO C57BL/6 mice were generated according to the protocol described in Sebastián et al. (2012).

SH‐SY5Y, HeLa, and HEK293T SIRT6KO or WT control cells were generated according to the protocol described in Kaluski et al. (2017).

### 4.2 Analysis of brSIRT6KO Brain Transcriptomics

The brSIRT6KO mouse brain transcriptomic dataset was already published under GSE221077 accession. The analysis was as previously described in Smirnov et al. (2023). Briefly, raw sequencing reads of WT and brSIRT6KO mouse replicates were processed using *nf‐core*/*rnaseq* pipeline (v3.0) (Ewels et al. 2020) to gene expression estimates in ‘star_salmon’ mode using ‘mm39’ reference genome assembly, followed by differential expression analysis performed via *DESeq2* (Love et al. 2014) with significance thresholds set at an FDR *p*‐value < 0.05 and |log_2_(Fold Change)| > 0.58. KEGG enrichment analysis was performed using the ‘enrichKEGG’ function from the *ClusterProfiler* R package (Yu et al. 2012) with a threshold for hypergeometric FDR *p*‐value < 0.05.

Here, we selected only categories and genes that are relevant specifically to proteostasis, brain activity, and neurodegeneration and presented them. The supplementary tables include the full lists of genes and categories, with the categories of interest being highlighted.

### 4.3 Comparative Analysis With Human Transcriptome Portraits of AD

To compare functional changes in brSIRT6KO mice with those observed in AD, we downloaded human AD portrait dataset published in Hill and Gammie (2022). This AD portrait geneset was generated by the authors using 22 individual datasets (listed in the “AD portrait datasets” below), comprising gene expression profiles from AD and control human donors. Gene *p*‐value estimates for the comparisons between AD and controls from each individual dataset were utilized for the integrative scoring approach (Hill and Gammie 2022; Gammie 2022), resulting in the list of dysregulated genes (17,938 human genes in total), ranging from the most consistently dysregulated to the least affected. Each gene also has a corresponding score, reflecting both the magnitude of the contribution to AD changes and the directionality of change (positive scores indicate up‐regulated genes in individual AD datasets and negative scores correspond to the consistently downregulated genes) (Hill and Gammie 2022).

Using these genes and their sorted scores, we performed GSEA pathway analysis with the “gseKEGG” function from ClusterProfiler *ClusterProfiler* (Yu et al. 2012). The same analysis was conducted on the list of mouse brSIRT6KO transcriptomic changes (13,433 genes in total), sorted based on the signed −log_10_ transformed *p*‐values. This yielded the most significantly affected KEGG pathways (FDR *p*‐value < 0.05). Overlapping pathways from both datasets were then visualized based on GSEA normalized enrichment scores. The significance of pathway overlap between datasets was calculated via hypergeometric test in R. GSEAplots of the selected pathways were generated using ‘gseaplot2’ function from *enrichplot* package [Yu G (2024). enrichplot: Visualization of Functional Enrichment Result. R package version 1.24.4, https://yulab‐smu.top/biomedical‐knowledge‐mining‐book/].

### 4.4 AD Portrait Datasets

The list of human datasets used by Hill and Gammie (2022) (Hill and Gammie 2022) to create AD gene expression portrait is presented in the table below (taken from Table S1 of the original study):

**Table:** 

File	GEO dataset	Sex	Region
a281f	GSE5281	Females	Multiple brain regions*
a281m	GSE5281	Males	Multiple brain regions*
a422b96	GSE84422	Both	Multiple brain regions*
a422b97	GSE84422	Both	Multiple brain regions*
a350f	GSE48350	Females	Multiple brain regions*
a350m	GSE48350	Males	Multiple brain regions*
a553f	GSE118553	Females	Multiple brain regions*
a553m	GSE118553	Males	Multiple brain regions*
a063f	GSE122063	Females	Multiple brain regions*
a063m	GSE122063	Males	Multiple brain regions*
a80f	GSE36980	Females	Multiple brain regions*
a80m	GSE36980	Males	Multiple brain regions*
ad9h	GSE132903	Females	Medial temporal cortex
ad10h	GSE132903	Males	Medial temporal cortex
ad11h	GSE33000	Females	Prefrontal cortex
ad12h	GSE33000	Males	Prefrontal cortex
ad51h	GSE1297	Both	Multiple brain regions*
ad64h	GSE29378	Both	Multiple brain regions*
ad65h	GSE29378	Both	Multiple brain regions*
ad66h	GSE44771	Females	Visual cortex
ad67h	GSE44771	Males	Visual cortex
ad71h	GSE53697	Both	Cortex

* Regions are shown under individual datasets.

### 4.5 Cell Culture

All cells were grown in DMEM (catalog number: 41965039, Thermo‐Fisher Gibco, MA), supplemented with 1% L‐glutamine (catalog number: 25030024, Thermo‐Fisher Gibco, MA), 1% Penicillin/Streptomycin antibiotics mix (catalog number: 15140122, Thermo‐Fisher Gibco, MA), and 10% FBS (catalog number: 12657‐029, Thermo‐Fisher Gibco, MA). Incubation was 37°C, 5% CO_2_.

### 4.6 Chromatin‐Bound Protein Acid Extraction

The chromatin‐bound proteins were extracted from brain tissues and cell culture dry pellets according to the following protocol:

Brain tissues and cell culture pellets were resuspended in cytoplasmic protein lysis buffer (described below), in a volume equivalent to 3 times the volume of the tissue. For tissues, the samples were homogenized for 2 cycles of 30 s in an electric homogenizer; for cell pellets, the samples were homogenized by thorough pipetting. Once homogenized, samples were kept on ice for 20 min, then centrifuged for 10 min, 21,100 g, 4°C. The supernatant, which contains the non‐chromatin‐bound fraction of proteins, was transferred into new tubes.

The pellets (which contain the chromatin and cell leftovers) were washed twice with the same volume of cytoplasmic protein lysis buffer, incubated 5 min on ice and centrifuged for 5 min, 21,100 g, 4°C. then, Add 0.2 N HCl solution to the dry pellet (equivalent of 1/8–1/10 of the original cytoplasmic protein lysis buffer volume) and pipette thoroughly. Samples were incubated on ice for 20 min with occasional vortexes, then centrifuged for 10 min, 21,100 g, 4°C. Supernatants (contain the chromatin‐bound proteins) were transferred to new tubes and neutralized by adding 1 M Tris pH 8 (the same volumes as the 0.2 N HCl), then vortexed. Protein concentrations were determined using Bradford assays.

cytoplasmic protein lysis buffer: 10 mM HEPES pH 7.4, 10 mM KCl, 0.05% NP‐40, phosphatase inhibitor cocktail X1 (APExBIO K1013), 0.2 mM PMSF, 0.0015 mM trichostatin A.

### 4.7 Total Protein Extraction

Total protein extraction from brain tissues and cell culture dry pellets was done according to the following protocol:

Brain tissues and cell culture pellets were resuspended in RIPA lysis buffer, in a volume equivalent to 3 times the volume of the tissue. For tissues, the samples were homogenized for 2 cycles of 30 s in an electric homogenizer; for cell pellets, the samples were homogenized by thorough pipetting. Once homogenized, samples were kept on ice for 20 min, then centrifuged for 30 min, 21,100 g, 4°C. The supernatant, which contains the extracted proteins, was transferred to a new tube and protein concentrations were determined using Bradford assays.

### 4.8 Antibody List

**Table:** 

Antibody	Company	Catalog number	Dilution
TIP5	Abcam	ab195278	1:1000
H3K56ac	Active Motif	39281	1:3000
H3	Santa Cruz Biotechnology	sc‐10809	1:3000
BrdU (FUrd)	Sigma‐Aldrich	B8434‐100UL	1:1000
Nucleolin	Abcam	ab22758	1:1000
SNF2H	Novus Biologicals	NB100‐55310	1:1000
Fibrillarin	Abcam	ab4566	1:1000
NeuN	Sigma‐Aldrich	FCMAB317PE	1:1000
Puromycin	Millipore	MABE343	1:12,000 WB and 1:10000 for IF
4EBP1‐phosphorylated	Cell Signaling Technology	CST9451	1:1000
HSPA9	Santa Cruz Biotechnology	sc‐133137	1:1000
HSPD1	Santa Cruz Biotechnology	sc‐13115	1:1000
HSP90β	Developmental Studies Hybridoma Bank	H90‐10	1:1000
CRYAB	Santa Cruz Biotechnology	sc‐22744	1:1000
DnaJB11	Proteintech	15484‐1‐AP	1:1000
HSPA2	Sigma‐Aldrich	HPA000798‐100UL	1:1000
GFP	Sigma‐Aldrich	11814460001	1:1000
SIRT6	Abcam	ab88494	1:1000
Rabbit anti‐mouse IgG H&L (HRP)	Abcam	ab97046	1:10000
Goat anti rabbit IgG H&L (HRP)	Abcam	ab6721	1:10000
Alexa Fluor 488 AffiniPure Donkey Anti‐Mouse IgG (H + L)	Jackson ImmunoResearch	715‐545‐150	1:250
Alexa‐Fluor 555 Donkey Anti‐Mouse IgG H&L	Abcam	ab150110	1:250
Alexa Fluor 488 AffiniPure Donkey Anti‐Rabbit IgG (H + L)	Jackson ImmunoResearch	711‐545‐152	1:250
Alexa Fluor 594 AffiniPure Donkey Anti‐Rabbit IgG (H + L)	Jackson ImmunoResearch	711‐585‐152	1:250

### 4.9 Plasmids and Transfections

TIP5‐GFP plasmid—addgene plasmid #65373 – https://www.addgene.org/65373/.

*Renilla* Luciferase vector—Promega pRL‐null.

Q74‐EGFP plasmid—addgene plasmid #40262 – https://www.addgene.org/40262/.

Q74‐EGFP plasmid—addgene plasmid #40261 – https://www.addgene.org/40261/.

Q0‐EGFP plasmid—pEGFP‐C1 backbone vector.

All other plasmids were homemade: shGFP, shSNF2H, empty vector (CMV‐flag backbone).

Transfections were conducted using PolyJet In Vitro DNA Transfection Reagent (catalog number: SL100688, SignaGen Laboratories, MD), according to the manufacturer's protocol.

### 4.10 4PBA Treatment

Media with 4PBA were prepared as previously described in Stein et al. (2022).

### 4.11 5‐Fluorouridine Nucleolar Transcription Labelling

Nucleolar transcription labeling was conducted as previously described in Portillo et al. (2021).

### 4.12 SUnSET Puromycin Labeling

The SUnSET (Schmidt et al. 2009) experiment was done as previously described in Stein et al. (2022). Briefly, cells were grown in regular growth conditions (see above) and plated in 6‐well plates. On the following day, puromycin was added to a final concentration of 10 μg/mL directly to the cell media, then the cells were re‐incubated in 37°C for 10–30 min, depending on the cell line (SH‐SY5Y—~18 min; HeLa—~25 min; HEK293T—~27 min). Once puromycin labeling was done, cells were collected for total protein extraction (see above).

For immunofluorescence, cells were incubated with 10 μg/mL puromycin for 45 min. In relevant experimental conditions, cycloheximide (CHX) was added 5 min prior to puromycin labeling. After incubation, the cells were washed three times with PBS and fixed with 4% paraformaldehyde (PFA). Immunofluorescence was performed using a primary antibody against puromycin (see antibody list) for 12 h at 4°C. In case 4PBA or transfections were conducted, they were done as previously described (Stein et al. 2022; see above), and the puromycin labeling was done 24‐48 h after the treatment/transfection.

Cycloheximide was used as a negative control, and was added 5 min before the puromycin labeling initiation, in a final concentration of 1–10 μg/mL.

### 4.13 Nucleolar Size and Intensity

For quantification, we used CellProfiler 4.2.4 (Stirling et al. 2021) to measure nucleolar intensities and size. Nucleoli were first segmented based on shape and intensity (using NCL or FBL markers). Adjacent nucleoli segmented as separate objects were merged into a single object named Nucleolus_individual. Next, we segmented the nucleus using the Hoechst channel, masking the nucleolus using the nucleus as a reference (Nucleolicombined). This enabled the quantification of the total nuclear area and intensity while eliminating objects outside the nucleus. A module was added to relate the nucleus to the nucleolus_individual, quantifying the total number of nucleoli per nucleus. Additional modules were included to measure intensity and area.

To verify segmentation, we asked CellProfiler to overlap each channel with the respective objects and masks, generating images for quality control. Only cells with accurate nucleus and nucleolus segmentation (validated by overlap images) were included in the statistical analysis, with inaccurately segmented cells excluded. A detailed spreadsheet with curated data was generated for each experiment. Similarly, the 5‐fluorouridine labeling pipeline was used, just adjusted by fluorescence channel and threshold.

### 4.14 Polysome Profiling

HeLa cells were plated in 15‐cm plates (~1.5 million cells per plate) and left to grow for 2 days. Then, cells were collected using trypsin (with supplemented medium neutralization) and washed with PBS. Cells were lysed with polysome profiling lysis buffer (50 mM HEPES pH 7.5, 100 mM potassium acetate, 15 mM magnesium acetate, 5% glycerol, 0.5% Triton X‐100, 50ug/ml cycloheximide, EDTA‐free protease inhibitor cocktail X1, 1 mM PMSF, 8 U/mL RNase‐free DNase) for 5′ on ice, followed by 5′ centrifuge (4°C, 2000 g, 5′). Lysate supernatants were loaded on 15%–60% sucrose gradients (buffer composition, apart from the sucrose: 50 mM HEPES pH 7.5, 100 mM potassium acetate, 10 mM magnesium acetate, 25ug/ml cycloheximide), and then ultracentrifuged (SW40Ti rotor, 4°C, 35000RPM, 3.5 h). Spectra were read using BioComp Triax Flow Spectrometer machine.

To allow a proper and normalized comparison between the samples, with no interference of intervening variables among the samples (such as the number of measured cells, the volume of lysate etc.), the OD_260_ spectra were normalized for each sample: the local minimum between the large subunit (60S) and the monosome (80S) was found and defined as the first point (X = 0). The OD_260_ reads were normalized to both the maximum and minimum values of each sample (so Y = 0 is the minimal OD_260_ and Y = 1 is the maximum OD_260_). The X‐axis was rescaled between the first and last datapoints on the graphs.

The monosome fraction value is the sum of all the Y values from X = 0 to the local minimum between the monosome and the disome (the first peak after the monosome), and the polysome fraction value is the sum of all the Y values from this local minimum to X = 100 (the last datapoint). The ribosomal activity of each sample was defined as the ratio between the polysome and the monosome fractions.

To avoid any RNA degradation, all procedures were done with RNase‐free reagents and equipment.

### 4.15 Aggregate Measurements by Immunofluorescence

#### 4.15.1 Defining Nuclei and Cells

Cells were plated and transfected as described above, and the micrograph analysis was conducted using CellProflier (Stirling et al. 2021). Nuclei were defined using Hoechst staining and the ‘IdentifyPrimaryObject’ module. Then, the cell cytoplasm of each nucleus was defined using the ‘IdentifySecondaryObject’ module applied on the polyQ fluorescent channel with the nuclei as input objects, as the GFP signal was spread throughout the entire or nearly entire cell.

#### 4.15.2 Automatic Aggregation Quantification

The principle behind the automatic aggregation measurement is that aggregated polyQ‐EGFP proteins create relatively bright areas compared to their surroundings (which have less EGFP signal and are hence darker), while non‐aggregated polyQ‐EGFP will be evenly distributed throughout the cell. This leads to a ‘rough’ texture of the EGFP signal in cells with aggregated proteins (i.e., more transitions from bright to dark areas inside the cell). In contrast, cells with non‐aggregated proteins will have a ‘smoother’ texture (i.e., larger areas with similar brightness inside the cell). In CellProfiler, “texture” refers to how evenly or unevenly the pixel intensities are distributed inside an object.

Therefore, within each cell, we quantified the distribution of the polyQ‐EGFP signal throughout the cell using the ‘MeasureTexture’ module. From the available texture parameters, we used the ‘contrast’ value, as it best captures local variation in signal intensity. Cells with less aggregation will have a more uniform signal, which results in low contrast values; while aggregates create areas of high intensity next to low intensity, leading to higher contrast values.

#### 4.15.3 Manual Aggregation Scoring

The micrographs were inspected manually, and each cell was assigned with a score of S0–S2. Cells without aggregates or with smooth texture were scored as S0, cells with 1–5 aggregates or medium texture as S1, and cells with more than 5 aggregates or rough texture as S2.

### 4.16 Luciferase Assays

#### 4.16.1 Heat Shock Luciferase‐Based Assays

20 k cells were plated in 48‐well plates and 24 h post‐plating, transfected with Renilla plasmid (10%), supplemented with an empty vector (see above). 24 h after transfections, cells were put in 42°C, 5% CO_2_ incubator for an hour, then in 37°C recovery for 90–120 min.

Lysis, luciferase substrates and luminescence readings were conducted using the Promega *Renilla* Luciferase Assay System kit (Promega E2820), according to the manufacturer's protocol. Protein refold capacity was calculated as the division of the *Renilla* activity after heat shock‐recovery in non‐shocked activity. Importantly, since SIRT6‐deficient cells present elevated translation, WT and KO cells were normalized to their own non‐heat‐shocked samples.

### 4.17 Nematode Strains and Growth Conditions

Strains used in this work included N2 (WT) and AM101: *rmIs110[pF25B3.3::Q40::YFP]* (Gidalevitz et al. 2006), available from the *Caenorhabditis* Genetics Center, and *sir‐2.4*KO, PHX1190: *sir‐2.4(syb1190)*, a 1428 bp CRISPR deletion of exons 2–5 (SunyBiotech). Mutant strains were outcrossed into our N2 stock (*n* ≥ 3)—∆*sir‐2.4*, DT11 is a genetic cross between AM85 × PHX1190—nQ40::YFP; ∆*sir‐2.4*. Standard genetic crossing techniques were used to construct mutant strains, and single‐worm PCR (Phire Animal Tissue Direct PCR Kit, Thermo Scientific) was used to verify the mutations as previously described (Meshnik et al. 2022). Nematodes were grown on NGM plates seeded with the *Escherichia coli* OP50‐1 strain and maintained at 20°C under standard conditions (Wood, W. B. The Nematode *Caenorhabditis elegans* . *Cold Spring Harb. Monogr. Arch. Vol. 17 Nematode* *Caenorhabditis elegans* (1988).) unless stated otherwise. For the 4PBA assays, plates were supplemented with 4PBA (Sigma). Age‐synchronized animals were obtained by placing adults on fresh plates to lay eggs for 3–5 h (Dror et al. 2020). Unless otherwise stated, eggs, laid at 15°C, were grown at 25°C for the duration of an experiment. The first day of adulthood is set before the onset of egg‐laying (young adults). To avoid progeny contamination, animals were moved to fresh plates during the reproductive period.

### 4.18 Visualization of *C. elegans* Nucleoli

To assess nucleoli size upon depletion of *sir‐2.4*, a genetic cross with a nucleolar marker strain (COP262) has been conducted. Three cohorts of each 11–12 individual nematodes of strains COP262 (*sir‐2.4* wild type) and JKM243 (*sir‐2.4* knockout) were analyzed. In brief, day 4‐old nematodes were mounted on 2% agarose pads and anesthetized with 250 mM NaN_3_. Fluorometric imaging was performed on an epifluorescent microscope (Axio Imager. Z2; Zeiss, Oberkochen, Germany) at 630‐fold magnification (Plan Apochromat 63/1.40 Oil DIC M27 objective). GFP was excited at 488 nm, lamp intensity set to 80%, and acquisition time was set to 100 ms.

Images were analyzed in ‘Fiji’ (Schindelin et al. 2012), measuring nucleolar area [μm (Bertram and Tanzi 2005)] in the nuclei of the two most anterior intestinal cells of the nematodes.

### 4.19 Thermotolerance Test

To assess paralysis after heat shock, day 1 adult and day 2 adult nematodes were incubated at 37°C for 1–2 h on NGM‐plates. Paralysis was tested by prodding worms with a platinum wire. Recovery was scored by monitoring motility 4 h after the heat shock. Three cohorts were tested (total of *n* > 30 per strain per day).

### 4.20 Motility Assay

For thrashing rates, age‐synchronized animals were monitored, and thrashes (changes in bending direction at mid‐body) were counted, as in (Dror et al. 2020). Values are presented as bends per minute. 500 μL of 33 mM 4‐phenylbutyric acid were dropped on 6 cm NGM plates and air dried (final concentration ≈1.65 mM). 10–15 L4 larvae were placed on the prepared plates and grown for 24 h. To count thrashing, nematodes were transferred to M9 medium, given 3 min acclimation time, and thrashing was counted manually for 20 s. Three independent cohorts of each 12 nematodes were analyzed.

### 4.21 Puromycin Labeling in Worms

30 adult worms were transferred into SUnSET buffer (83.9 mM Tris pH 7.4, 40 mM NaCl, 5 μ/mL cholesterol, OP50‐1 bacteria, and 10 μg/mL puromycin) and incubated for 4–8 h (the same timeframe for all conditions in each experiment) at 25°C with gentle shaking (200 RPM). The incubation was followed by a brief spin down, and the supernatant was discarded. Laemmli buffer (×4) was added to the pellet (worms only) and heated to 95°C with shaking (1000 RPM) for 5 min. Samples were detected using western blot analysis.

### 4.22 Motility Decline Assay

To monitor thrashing activity over aging, age‐synchronized nematode cultures were grown at 25°C to day 1, 3, and 5 of adulthood and placed in M9‐Medium to count thrashes. After 3 min acclimation time, thrashing was counted manually for 30 s. Three independent cohorts of each 20 nematodes were analyzed.

### 4.23 Lifespan Assay

To monitor the survival of nematodes upon 4PBA treatment, nematodes were age synchronized and placed in groups of 30–40 animals on 6 cm NGM plates and grown at 25°C. For 4PBA treatment, 500 μL of 33 mM 4‐phenylbutyric acid were dropped on 6 cm NGM plates and air dried (final concentration ≈1.65 mM) approx. 1 h prior to nematode passage. Survival was tested every day by prodding worms with platinum wire. Nematodes were passaged to fresh plates daily during their fertile period; afterwards, they were passaged every second day. Nematodes that died due to passaging were censored in the analysis. Analysis was done according to Kaplan–Meier; LogRank test was used to test for significant differences using GraphPad Prism 10. Two independent cohorts of each 72–118 nematodes were analyzed.

### 4.24 Statistics

Analyses were done as described in the supplementary statistics table.

## Author Contributions

D.S., M.P., S.K., A.G.‐V., Y.L., and M.E. performed all the cell culture work and the corresponding molecular experiments. D.S., M.P., A.G.‐V., and E.E. performed all the mouse‐related experiments. C.G. and D.S. performed all the *C. elegans* experiments. DiSm and D.S. performed the bioinformatical analyses. S.D. provided help with designing and planning the polysome profiling experiments. E.K. provided help with designing the bioinformatical analyses. C.G., A.B.‐Z., and B.S. provided help with designing, planning, analyzing, and interpreting the *C. elegans* experiments. D.T. and D.S. conceived, led, planned, and designed the study, as well as wrote the manuscript. All the authors reviewed the manuscript.

## Funding

The study was funded by the European Research Council (ERC) under the European Union's Horizon 2020 research and innovation program (grant agreement No 849029); The David and Inez Myers foundation; the Israeli Ministry of Science and Technology (MOST); The Israel Science foundation (No. 422/23); the High‐tech, Biotech and Negev fellowships of Kreitman School of Advanced Research of Ben‐Gurion University; Deutsche Forschungsgemeinschaft (DFG) grant number 540136447; EMBO Short‐Term Fellowship (STF 8688).

## Conflicts of Interest

The authors declare no conflicts of interest.

## Supporting information


**Data S1:** Supplementary tables ‐ statistics and RNA‐seq data ‐ acel70384‐sup‐0001‐Supinfo01.xlsx.

**Data S2:** Main and Supplementary figures ‐ acel70384‐sup‐0002‐Figures.pdf.

## Data Availability

Data sharing is not applicable to this article as no new datasets were created in this study. Any previously published datasets and analyses that were used in this study are detailed in the Section 4.
