# Supplementary material for: SIRT6 Regulates Protein Synthesis and Folding Through Nucleolar Remodeling
Source: Aging Cell. 2026 Feb 17;25(2):e70384. doi: 10.1111/acel.70384 (PMC12913217; doi:10.1111/acel.70384)
Supplement: Supplementary file 2 — Data S2: Main and Supplementary figures ‐ acel70384‐sup‐0002‐Figures.pdf. [file ACEL-25-e70384-s001.pdf]

Graphical Abstract

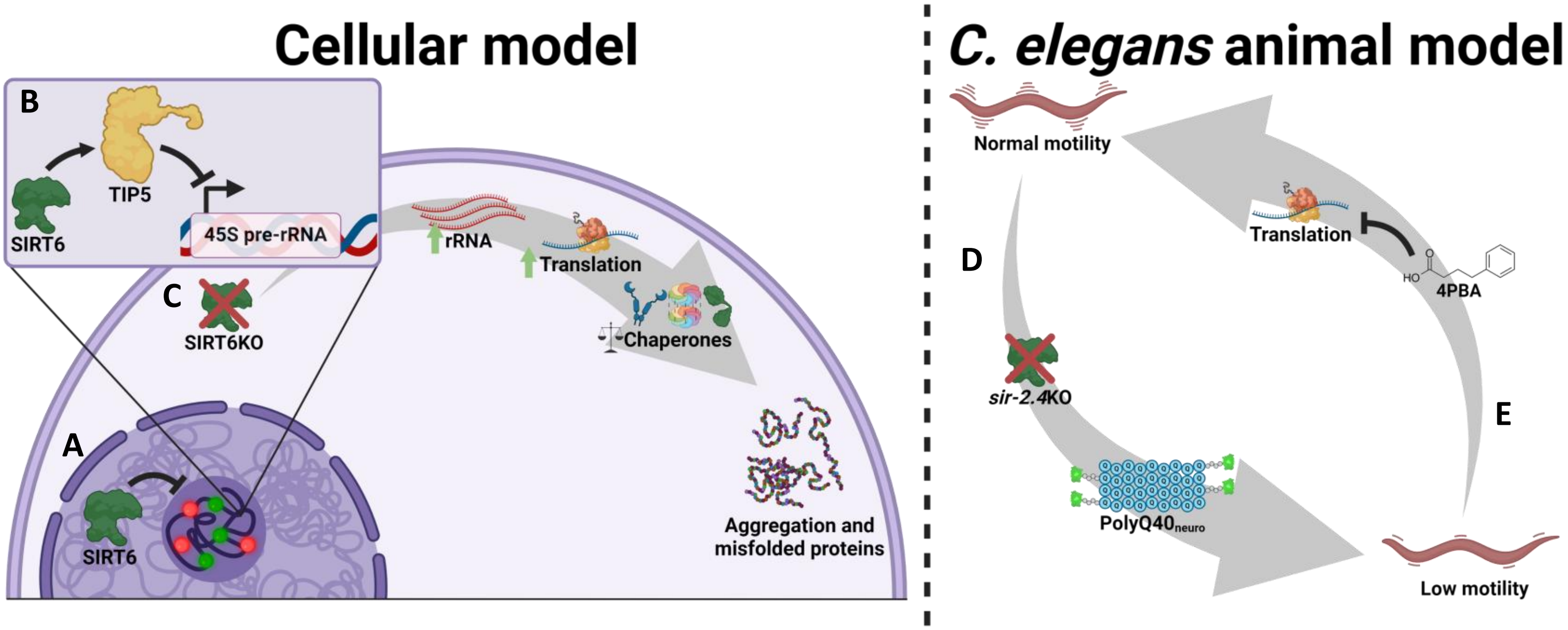

Graphical Abstract. SIRT6 regulates proteostasis through nucleolar regulation. SIRT6 regulates (A) pre-rRNA editing through nucleolar factors, as well as (B) rRNA transcription through TIP5 – and thus the NoRC – chromatin recruitment. (C) Once SIRT6 is depleted, the rRNA production and editing are increased, leading to a dramatic elevation in translation rates. However, chaperone levels remain unchanged, leading to the accumulation of misfolded proteins and aggregation upon SIRT6 deletion. (D) In a *C. elegans*-based model, deleting SIRT6 ortholog (*sir-2.4*) leads to impaired heat shock resistance, as well as reduced motility upon overexpression of neuronal aggregation-prone protein (PolyQ40$_{neuro}$). (E) However, attenuating translation using 4PBA leads to rescue of the *sir-2.4*KO;PolyQ40$_{neuro}$ strain.

Figure S1

A

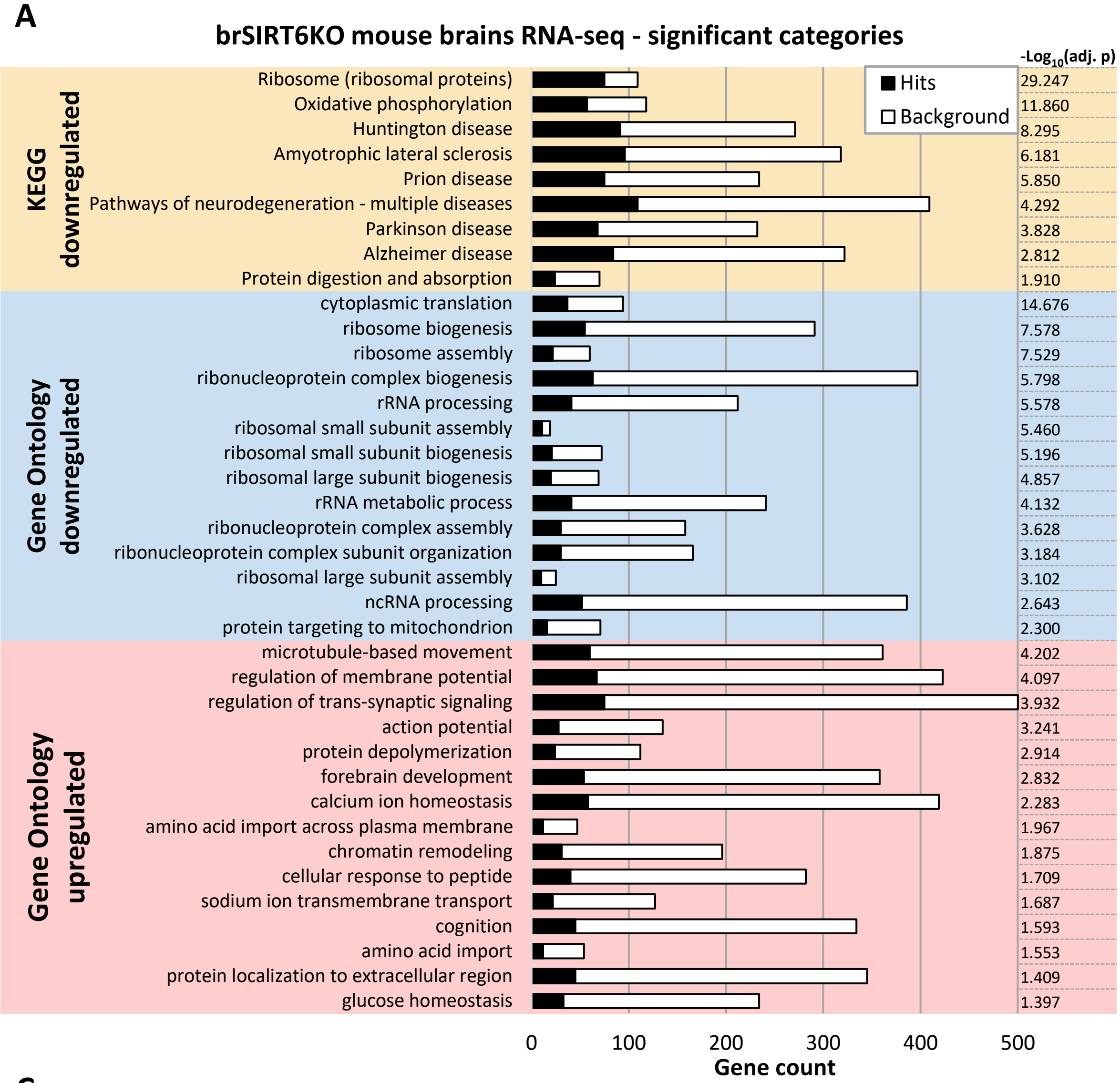

B

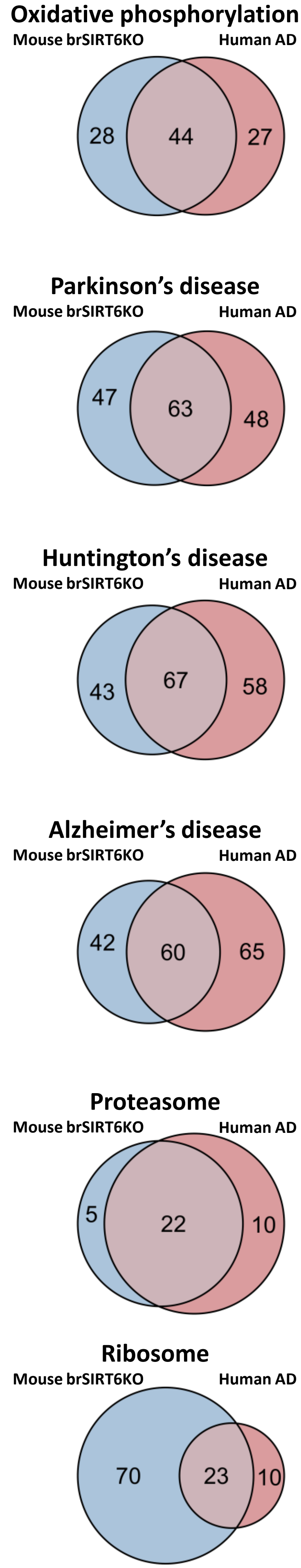

C

	Overlap	KO set size	AD set size	% overlap KO	% overlap AD	Jaccard	FDR p-value	Odds ratio
Oxidative phosphorylation	44	72	71	61.1	62	0.444	6.95E-86	672.059
Parkinson's disease	63	110	111	57.3	56.8	0.399	3.98E-106	322.882
Huntington's disease	67	110	125	60.9	53.6	0.399	3.32E-111	310.892
Alzheimer's disease	60	102	125	58.8	48	0.359	2.00E-97	254.402
Proteasome	22	27	32	81.5	68.8	0.595	1.43E-56	4323.141
Ribosome	23	93	33	24.7	69.7	0.223	1.75E-42	380.839

D

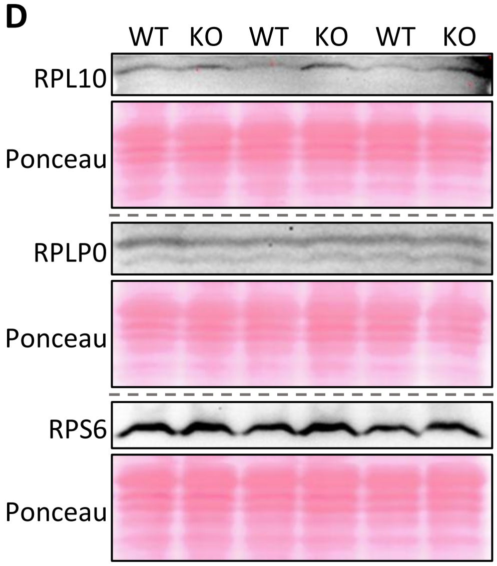

Figure S1. (A) Selected enriched KEGG and Gene Ontology categories from RNA-seq data of brSIRT6KO mouse brains, ranked by significance, with all categories showing an adjusted p-value < 0.05. Right-hand table includes the corresponding -log$_{10}$(adjusted p-value). The original analysis was performed in Smirnov et al. (2023). (B) Overlap of the genes changed in brSIRT6KO brains and human AD patient datasets, presented in Euler diagrams. (C) Summary table of the overlapping categories changing in brSIRT6KO brains and human AD patient datasets. (D) Representative western blots of ribosomal proteins in brains of WT and brSIRT6KO mice.

Figure S2

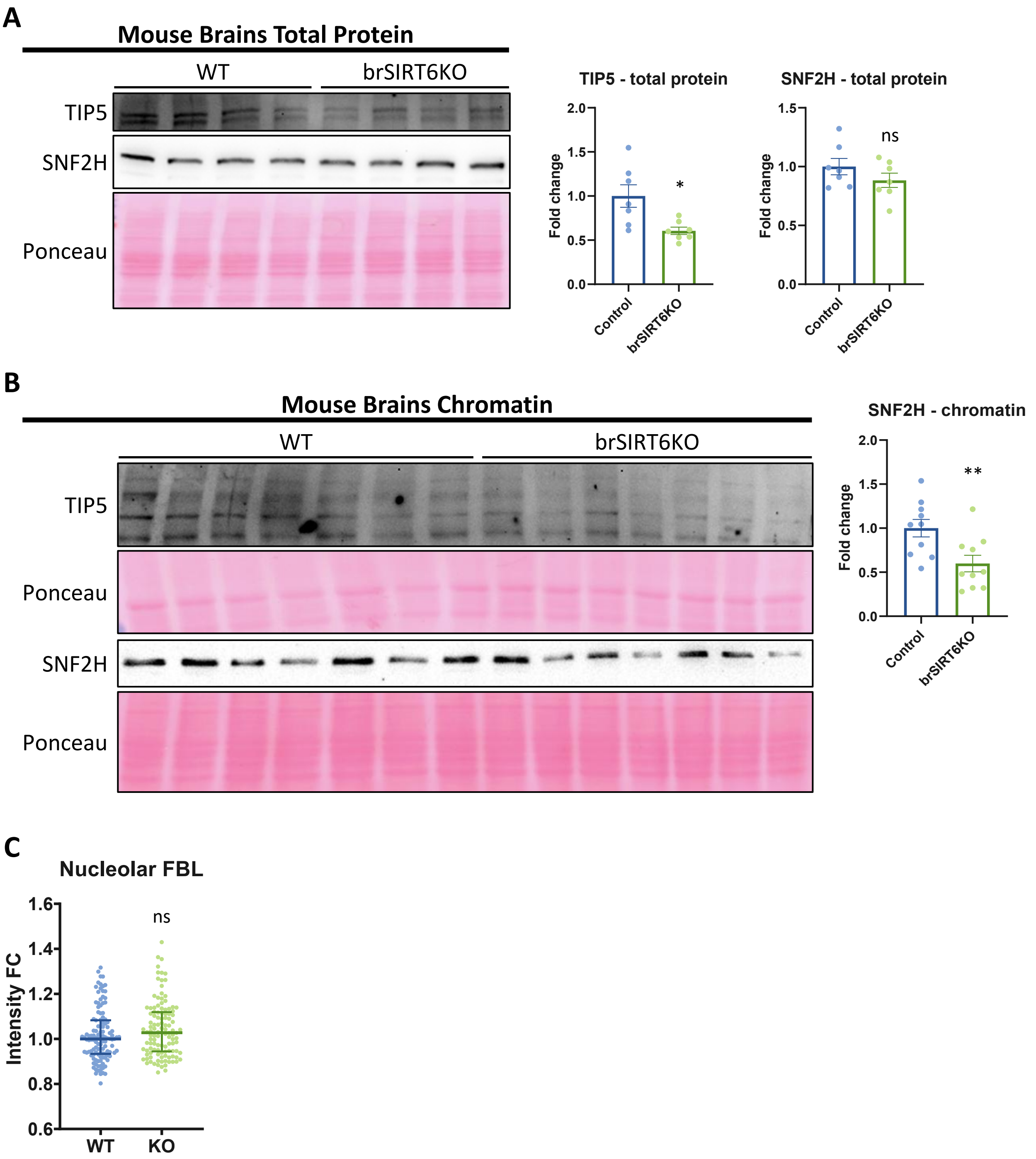

Figure S2. (A) Representative western blots of total TIP5/SNF2H proteins in brSIRT6KO brains. Left panel – representative blots; right panel – quantified total TIP5/SNF2H intensities, normalized to Ponceau total protein staining. (B) Representative western blots of chromatin-bound TIP5/SNF2H proteins in brSIRT6KO brains. Panels (left to right) – representative blots; quantified chromatin-bound TIP5/SNF2H intensities, normalized to Ponceau total protein staining. (C) Quantification of fibrillarin (FBL) median pixel intensity inside the nucleolus, of fig. 2D. FC – fold change. ns - $p>0.05$, * - $p<0.05$, ** - $p<0.01$.

Figure S3

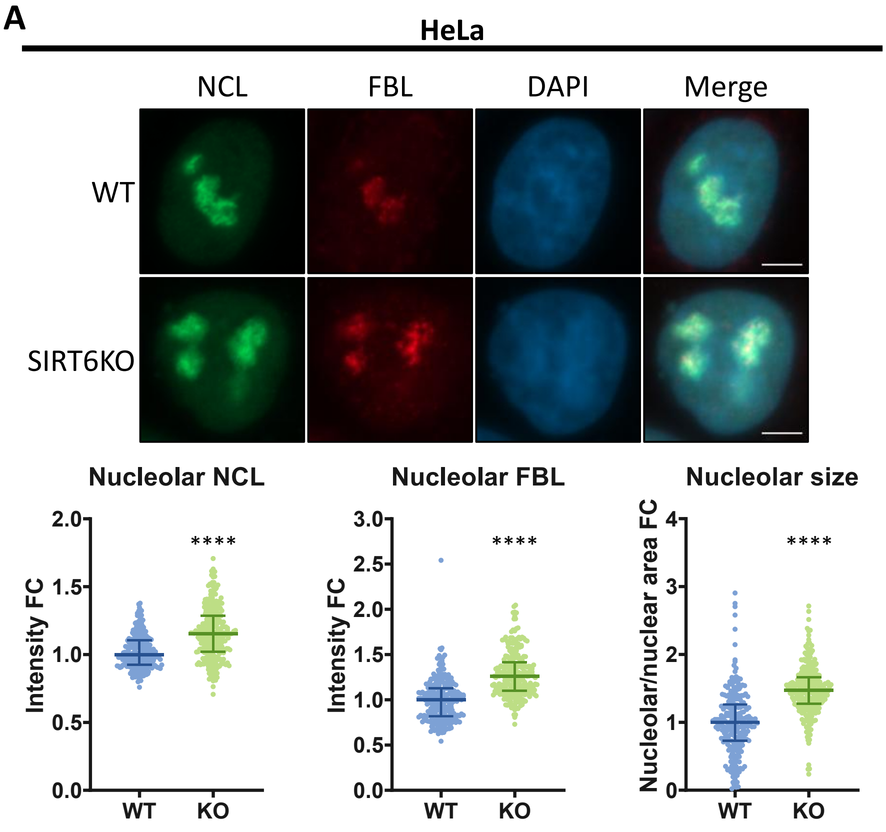

Figure S3. (A) Immunofluorescence of nucleolar markers in control and SIRT6KO HeLa cells. Panels (upper left to lower right) – representative photos; quantified nucleolar nucleolin (NCL) and fibrillarin (FBL) median intensities; nucleolar size fold change (nucleolar area/nuclear area ratio). n=518. Scale bars = 5µm. WT – control cells; KO – SIRT6KO cells. **** – $p<0.0001$.

Figure S4

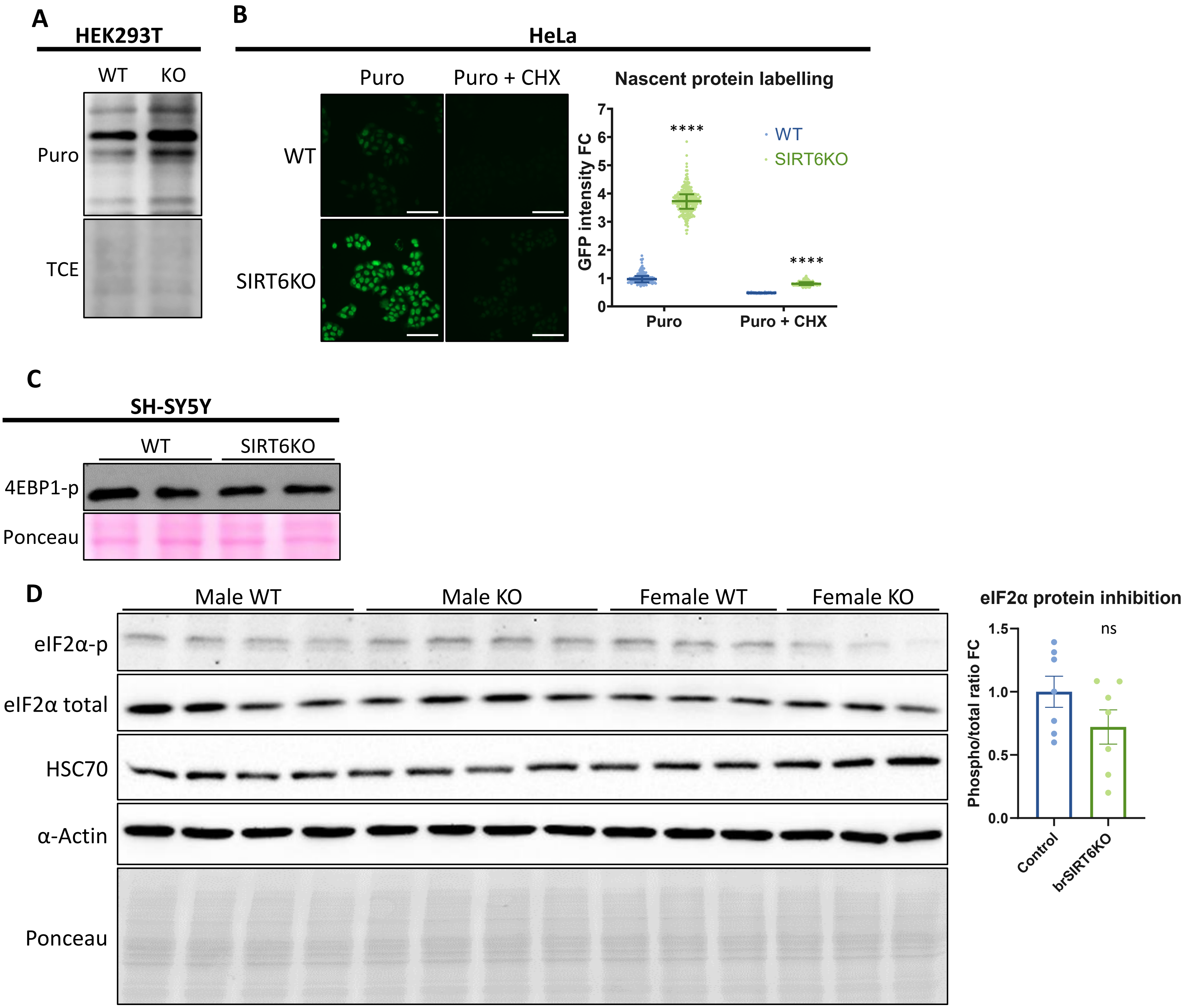

Figure S4. (A) A representative western blot of puromycin-based nascent protein labelling (SUnSET), in HEK293T cells. WT – control cells; KO – SIRT6KO cells; Puro – puromycin. (B) Immunofluorescence of puromycin-labelled nascent proteins in SIRT6KO and control HeLa cells. cells were incubated with 10 µg/ml puromycin for 45 minutes. Cycloheximide (CHX) was added 5 minutes prior to puromycin labeling. Left panel – representative photos; right panels – quantified puromycin intensity. Puro – puromycin; CHX – cycloheximide (negative control). Scale bars = 50µm. (C) A representative western blot of 4E-BP1 phosphorylation in SH-SY5Y cells. 4EBP1-p – 4E-BP1 phosphorylation. (D) A representative western blot of phosphorylated and total eIF2α in brain samples from WT and brSIRT6KO (KO) mice (both males and females). Panels (left to right) – representative blots; quantified eIF2α inhibition by phosphorylation, as calculated by the ratio between eIF2α phosphorylation and total protein levels. ns – p>0.05, **** – p<0.0001.

Figure S4 – continue

E

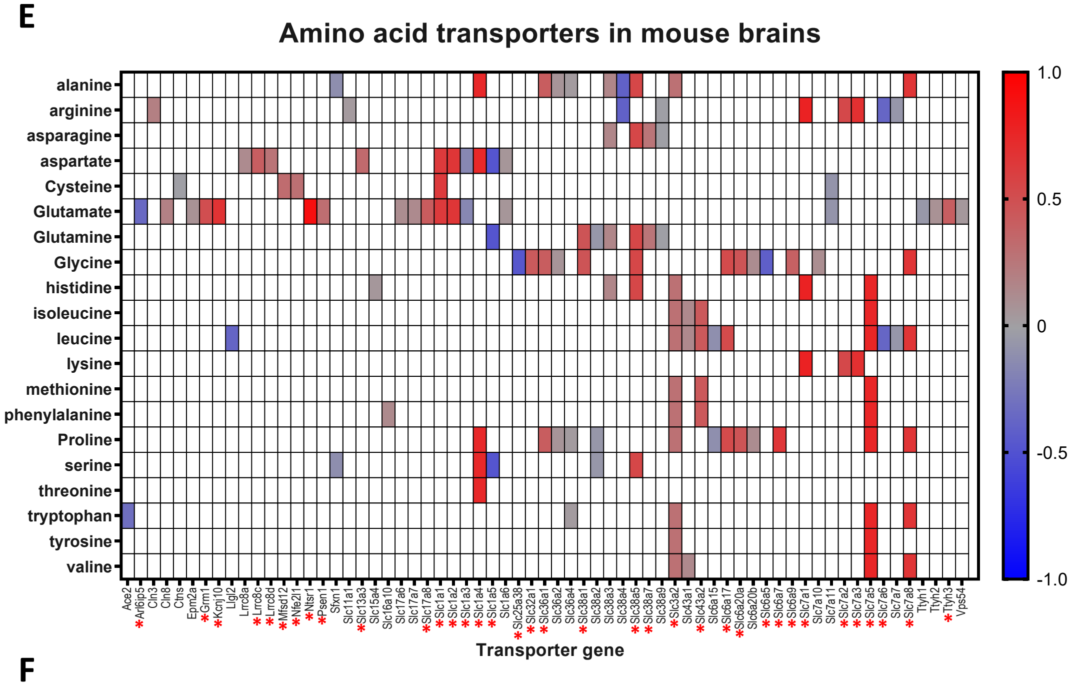

G

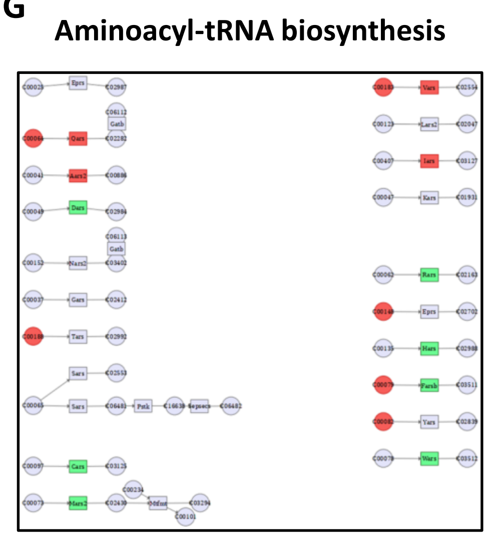

**Components**	74
**Metabolite hits**	6
**Gene hits**	12
**Raw p-value**	0.0008
**Holm adjusted p**	0.0647
**FDR**	0.0162
**Impact**	0.38356

F

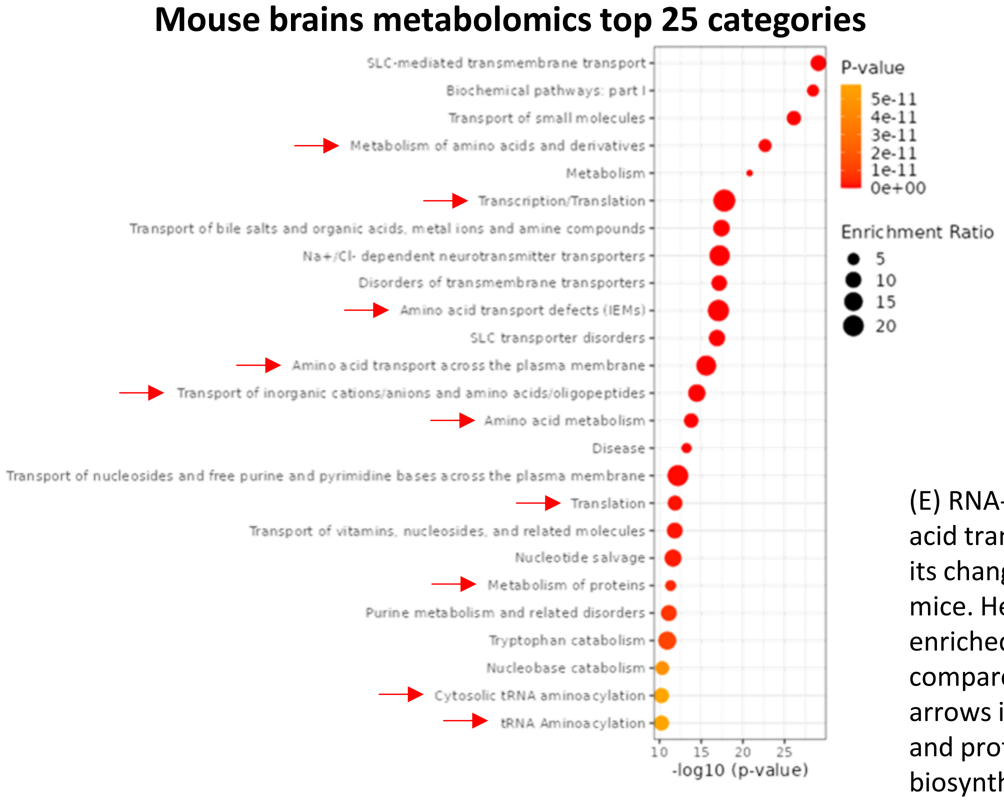

H

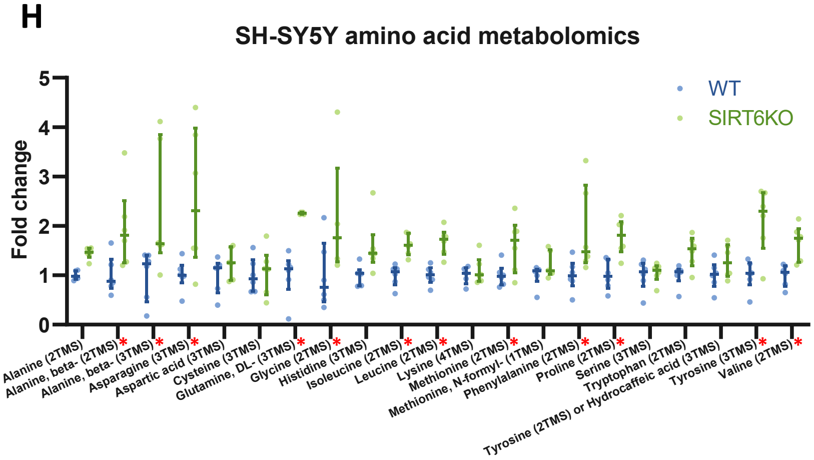

(E) RNA-seq of brSIRT6KO brains, presenting the amino acid transporter activity of each transporter gene, and its change in brain-specific SIRT6KO compared to WT mice. Heatmap represents $\log_2$(fold change). (F) Top 25 enriched categories of brSIRT6KO mouse brains compared to WT mice, in a metabolomics analysis. Red arrows indicate categories that are related to amino acid and protein metabolism. (G) Aminoacyl-tRNA biosynthesis pathway data, from the metabolite-gene joint analysis. Top panel – pathway map with significantly changed components marked. Bottom panel – a table with statistical parameters of the pathway. (H) Amino acid metabolomics in SIRT6KO/WT SH-SY5Y cells. Red asterisk – FDR<0.05.

Figure S5

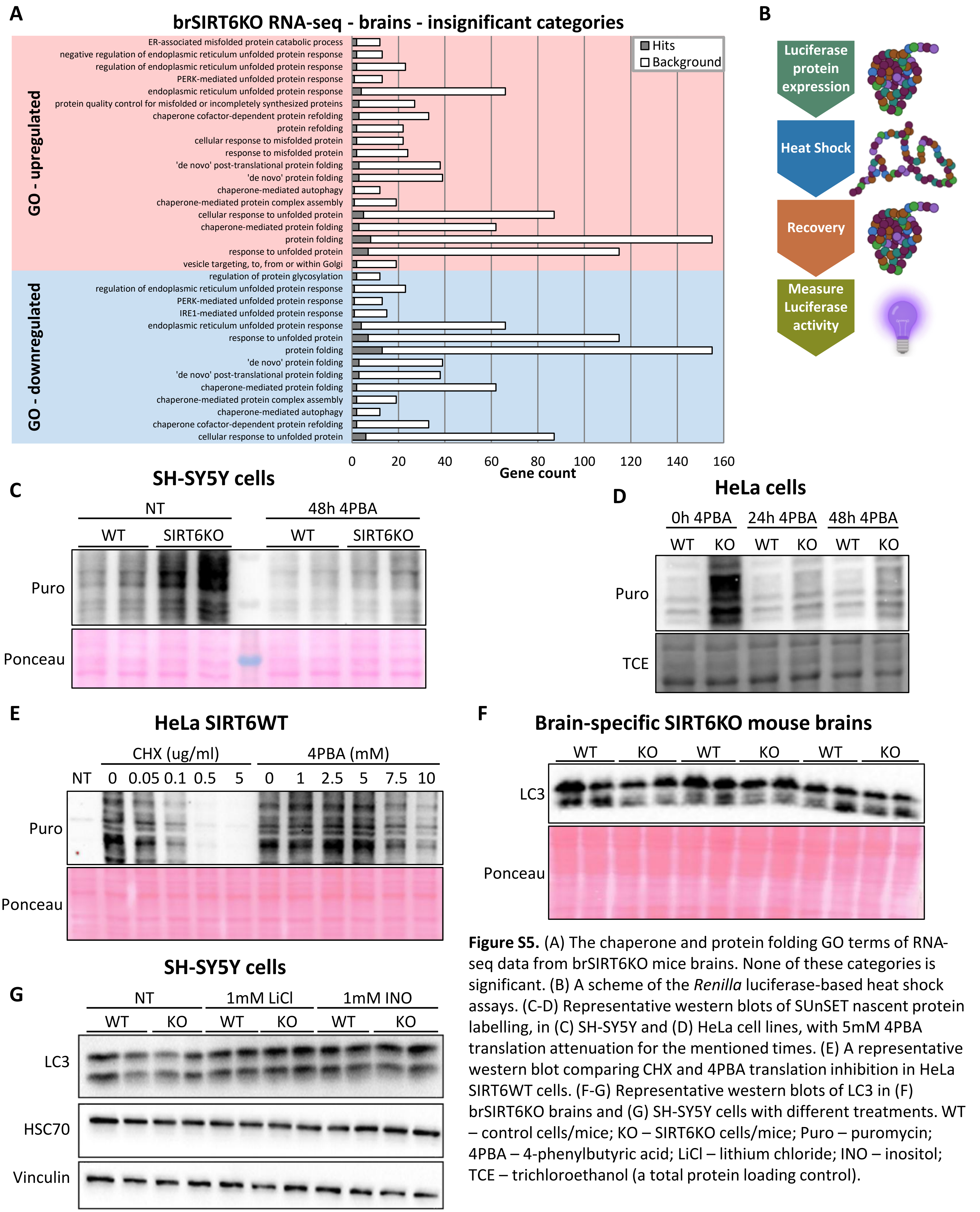

Figure S5. (A) The chaperone and protein folding GO terms of RNA-seq data from brSIRT6KO mice brains. None of these categories is significant. (B) A scheme of the *Renilla* luciferase-based heat shock assays. (C-D) Representative western blots of SUnSET nascent protein labelling, in (C) SH-SY5Y and (D) HeLa cell lines, with 5mM 4PBA translation attenuation for the mentioned times. (E) A representative western blot comparing CHX and 4PBA translation inhibition in HeLa SIRT6WT cells. (F-G) Representative western blots of LC3 in (F) brSIRT6KO brains and (G) SH-SY5Y cells with different treatments. WT – control cells/mice; KO – SIRT6KO cells/mice; Puro – puromycin; 4PBA – 4-phenylbutyric acid; LiCl – lithium chloride; INO – inositol; TCE – trichloroethanol (a total protein loading control).

Figure S6

A

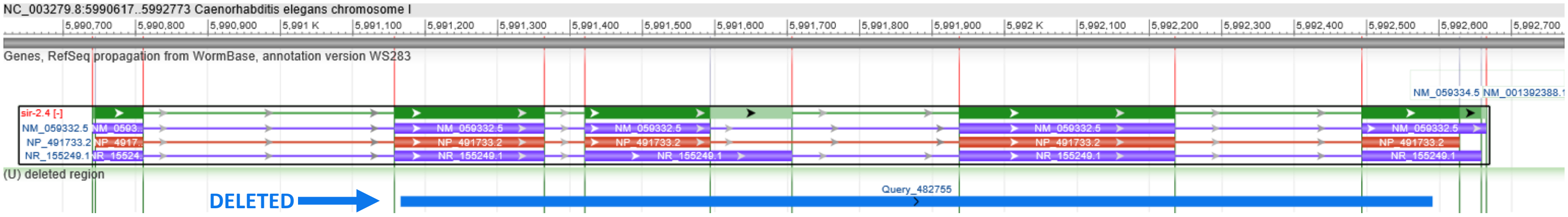

B

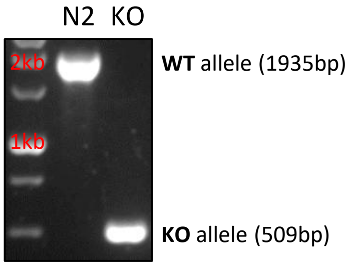

Figure S6. (A) *sir-2.4* locus in *C. elegans*. Deleted region is marked in blue and was done using CRISPR by SunyBiotech. (B) A representative PCR product of *sir-2.4* deletion validation.

Figure S7

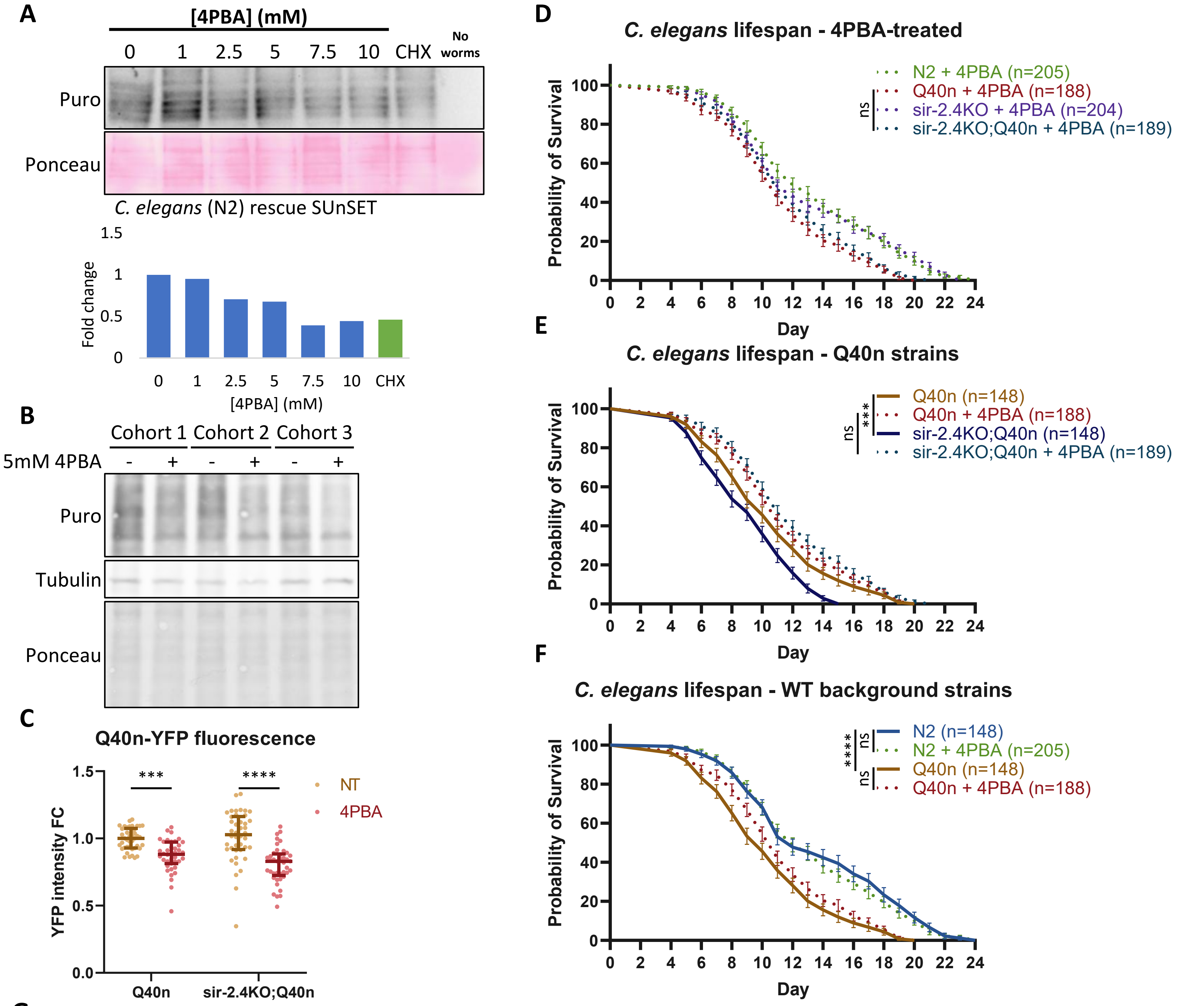

G

C. elegans survival - lifespan descriptive statistics		
strain	Median lifespan	Max lifespan
N2	13	22.5
N2 + 4PBA	12.5	32
Q40n	10	18.5
Q40n + 4PBA	10.5	19.5
*sir-2.4*KO	12	21
*sir-2.4*KO + 4PBA	11.5	22
Q40n;*sir-2.4*KO	9	14.5
Q40n;*sir-2.4*KO + 4PBA	11	19

C. elegans survival - statistics of lifespan medians			
Condition A	Condition B	Log-rank (Mantel-Cox) test p-value	Gehan-Breslow-Wilcoxon test p-value
N2	N2 + 4PBA	0.5626 (ns)	0.8426 (ns)
N2	Q40n	<0.0001 (****)	<0.0001 (****)
N2	*sir-2.4*KO	0.0119 (*)	0.1346 (ns)
N2	Q40n;*sir-2.4*KO	<0.0001 (****)	<0.0001 (****)
Q40n	Q40n +4PBA	0.0769 (ns)	0.0346 (*)
Q40n	Q40n;*sir-2.4*KO	0.0003 (***)	0.0091 (**)
Q40n + 4PBA	Q40n;*sir-2.4*KO + 4PBA	0.201 (ns)	0.232 (ns)
*sir-2.4*KO	*sir-2.4*KO + 4PBA	0.1137 (ns)	0.74 (ns)
Q40n;*sir-2.4*KO	Q40n;*sir-2.4*KO + 4PBA	<0.0001 (****)	<0.0001 (****)

Figure S7. (A) A representative western blot of 4PBA translation reduction SUnSET in day 1 of adulthood, including normalized quantifications. CHX – 50ug/ml cycloheximide; 'No worms' - the assay buffer with the inactive bacteria and a little living bacteria from the worms' plate without worms (negative control for bacterial background labelling). (B) A representative western blot of 5mM 4PBA translation reduction SUnSET in day 1 of adulthood, in 3 additional cohorts. (C) quantifications of YFP signal in *C. elegans* Q40n strains treated with 4-phenylbutyric acid (4PBA). n = 164. (D-F) Individual strains and treatments, separated for clarity. (G) *C. elegans* survival assay statistics summary of the lifespan data. Left table – median and maximal lifespans of each condition; right table – statistical tests done on the lifespan medians. ns – p>0.05, *** - p<0.001, **** – p<0.0001.
